# Subthalamic nucleus deep brain stimulation alleviates oxidative stress via mitophagy in Parkinson’s disease

**DOI:** 10.1038/s41531-024-00668-4

**Published:** 2024-03-06

**Authors:** Yingchuan Chen, Guanyu Zhu, Tianshuo Yuan, Ruoyu Ma, Xin Zhang, Fangang Meng, Anchao Yang, Tingting Du, Jianguo Zhang

**Affiliations:** 1 Department of Neurosurgery, Beijing Tiantan Hospital, Capital Medical University, 100070 Beijing, China; 2 Beijing Key Laboratory of Neurostimulation, 100070 Beijing, China; 3 Department of Functional Neurosurgery, Beijing Neurosurgical Institute, Capital Medical University, 100070 Beijing, China

**Keywords:** Parkinson's disease, Parkinson's disease

## Abstract

Subthalamic nucleus deep brain stimulation (STN-DBS) has the potential to delay Parkinson’s disease (PD) progression. Whether oxidative stress participates in the neuroprotective effects of DBS and related signaling pathways remains unknown. To address this, we applied STN-DBS to mice and monkey models of PD and collected brain tissue to evaluate mitophagy, oxidative stress, and related pathway. To confirm findings in animal experiments, a cohort of PD patients was recruited and oxidative stress was evaluated in cerebrospinal fluid. When PD mice received STN stimulation, the mTOR pathway was suppressed, accompanied by elevated LC3 II expression, increased mitophagosomes, and a decrease in p62 expression. The increase in mitophagy and balance of mitochondrial fission/fusion dynamics in the substantia nigra caused a marked enhancement of the antioxidant enzymes superoxide dismutase and glutathione levels. Subsequently, fewer mitochondrial apoptogenic factors were released to the cytoplasm, which resulted in a suppression of caspase activation and reservation of dopaminergic neurons. While interfaced with an mTOR activator, oxidative stress was no longer regulated by STN-DBS, with no neuroprotective effect. Similar results to those found in the rodent experiments were obtained in monkeys treated with chronic STN stimulation. Moreover, antioxidant enzymes in PD patients were increased after the operation, however, there was no relation between changes in antioxidant enzymes and motor impairment. Collectively, our study found that STN-DBS was able to increase mitophagy via an mTOR-dependent pathway, and oxidative stress was suppressed due to removal of damaged mitochondria, which was attributed to the dopaminergic neuroprotection of STN-DBS in PD.

## Introduction

Parkinson’s disease (PD), one of the most common neurodegenerative disorders, affects 2–3% of the world’s population over the age of 65^1^. The cardinal motor symptoms of the disease are the result of the selective degeneration of dopaminergic neurons in the substantia nigra (SN) pars compacta and other brain areas^2^. This results in motor symptoms, which include resting tremors, bradykinesia, rigidity, and postural instability, and non-motor symptoms, such as dementia and depression^3^.

The long-term use of levodopa, the gold standard of such therapies, is associated with severe side effects several years after the ‘honeymoon’^2^. Despite decades of research into finding a pharmacological cure, drugs for PD are merely symptomatic and do not halt the progressive loss of neurons^2^. Subthalamic nucleus deep brain stimulation (STN-DBS) has been identified as a very effective surgical therapy against the cardinal motor symptoms in patients with advanced PD^4^. Furthermore, early DBS reduces the need for and complexity of PD medications while providing long-term motor benefits over standard medical therapy and delaying disease progression^5–7^, which suggests that STN-DBS may have a neuroprotective effect^8^.

The pathogenic mechanism of PD is not fully understood; however, genetic factors and environmental exposures can contribute to its pathological progression, and mechanisms include changes in dopamine metabolism, mitochondrial dysfunction, endoplasmic reticulum stress, impaired autophagy, oxidative stress, and immunity^9–12^. Among the mechanisms mentioned above, oxidative stress plays an indispensable role. The generation of reactive oxygen species (ROS) is a normal physiological process and is essential for the survival of aerobic organisms. Nevertheless, the excessive production of ROS results in oxidative stress, causing damage to biomolecules, such as DNA and proteins, and apoptosis. A previous study has found that levels of superoxide dismutase (SOD), an important antioxidant enzyme, were lower in PD patients, furthermore, SOD level is negatively correlated with the severity of PD symptoms^13^. The intrinsic apoptotic pathway could be triggered by oxidate stress via generalized and irreversible mitochondrial outer membrane permeabilization^14^. Additionally, Cu_x_O nanoparticle clusters, an inorganic nanomaterial that functionally mimics the activities of peroxidase and SOD, further eliminated ROS and inhibited neurotoxicity in a model of PD^15^. Recent studies have found that damaged mitochondria undergo mitophagy to prevent the further spread of oxidative damage^16^. Deferiprone, an iron chelator, has undergone Phase II clinical trials, with observed improvements in motor function and improved quality of life without significant side effects. Iron chelators can reduce oxidative stress, and this may be attributed to the triggering of mitophagy^17^.

Due to the positive therapeutic effects of STN-DBS, many studies have focused on its mechanism. A recent study observed that DBS could inhibit or reverse the reduction in mitochondrial volume and numbers caused by PD^18^. Moreover, STN stimulation could rescue loss of dopaminergic SN neurons in a PD model^8^. Our previous study found that STN-DBS could exert neuroprotective effects against 6-OHDA-induced cell injury in PD by inducing autophagy^19^. However, the mechanism behind STN-DBS remains unclear and, specifically, whether it enhances neuroprotection by mitophagy-mediated oxidative stress needs to be illustrated. Several important structures in the basal ganglia are implicated in PD progression, and these structures have major differences in non-human primates (NHP) and rodents^20^. Given that NHPs are more similar to humans, in addition to rodents, monkeys and human specimens were included in the present study. It was found that STN-DBS was able to increase mitophagy via an mTOR-dependent pathway, and oxidative stress was suppressed. Consequently, apoptogenic factors released from mitochondria were reduced, which was attributed to the dopaminergic neuroprotection of STN-DBS in the SN of PD brains.

## Results

### Time point selection of STN-DBS

**Fig. 1:**
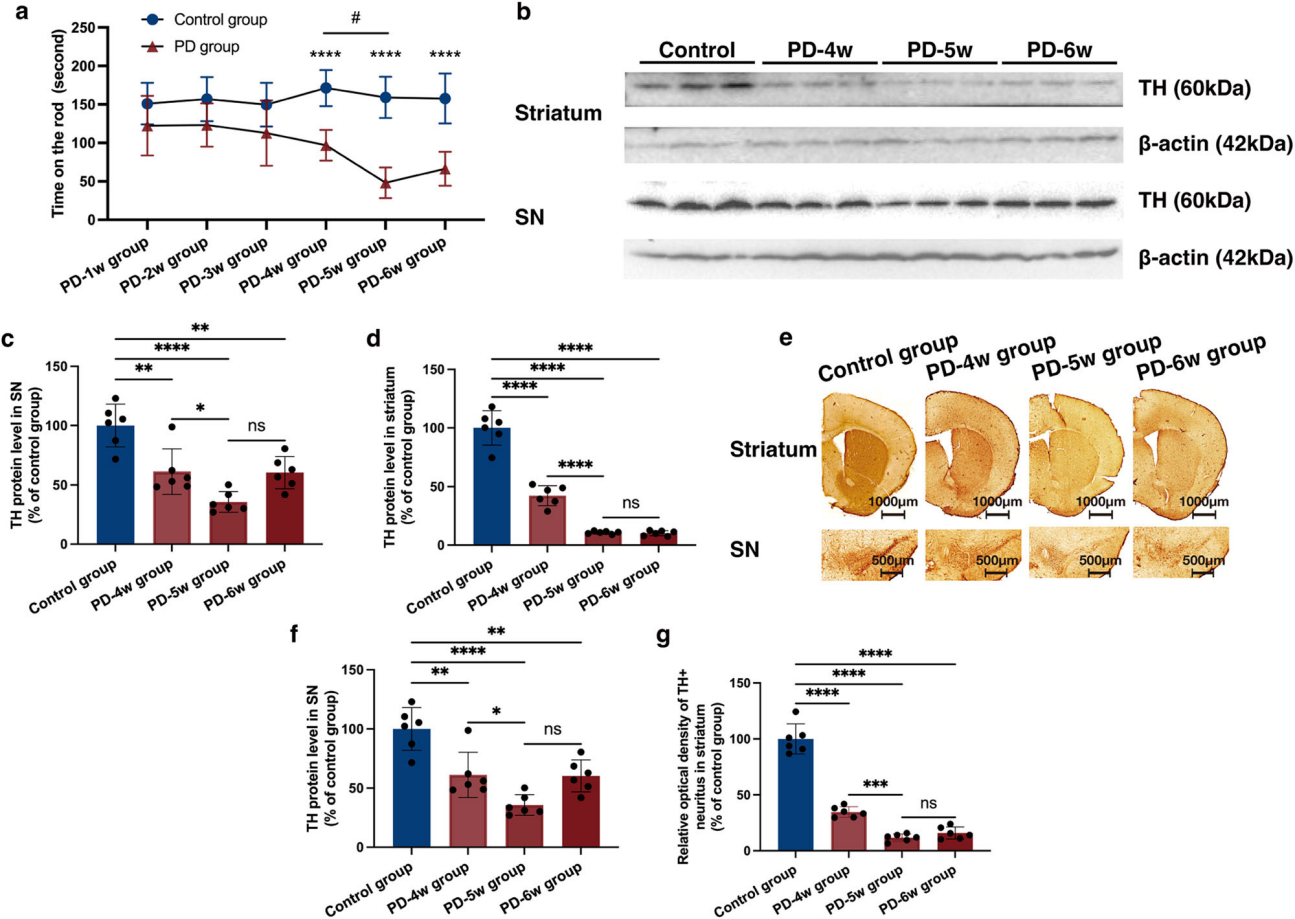
Time point selection of STN-DBS implantation. **a** The rotarod test of PD and normal animals at different time points. An obvious motor impairment was observed four weeks after model establishment, with a progressive impairment at four to five weeks. ^****^P < 0.0001, PD vs. control group; ^#^*P* < 0.05, the different time point comparison in the PD group (*n* = 8 per time point per group; F_Time(5,84)_ = 4.210, *P* = 0.0018; F_Group(1,84)_ = 112.6, *P* < 0.0001; two-way ANOVA followed by a Tukey post-hoc correction). **b** Western blot analysis of TH expression at different time points. **c**, **d** TH levels were significantly decreased four weeks after model establishment, with a progressive decline (*n* = 6 per group; Striatum: F_(3,20)_ = 143.6, *P* < 0.0001; SN: F_(3,20)_ = 17.84, *P* < 0.0001; one-way ANOVA followed by a Tukey post-hoc correction). **e** Immunohistochemistry staining of TH at different time points. **f**, **g** The TH^+^ neurons and neurites in the SN and striatum were significantly less detected four weeks after model establishment, and a progressive decline was found between four and five weeks after model establishment (*n* = 6 per group; TH^+^ neurons: F_(3,20)_ = 79.56, *P* < 0.0001; TH^+^ neurites: F_(3,20)_ = 165.2, *P* < 0.0001; one-way ANOVA followed by a Tukey post-hoc correction). ^*^P < 0.05; ^**^P < 0.01; ^***^P < 0.001; ^****^P < 0.0001. Error bars: standard deviation of the mean. STN-DBS subthalamic nuclei deep brain stimulation, PD Parkinson’s disease, SN substantia nigra, TH tyrosine hydroxylase, ns not significant.

To select the suitable time point for the lead implantation in mice, first, we evaluated the change in dopaminergic neuron loss along with time in the 1-methyl-4-phenyl-1,2,3,6-tetrahydropyridine (MPTP) model. The motor impairment was measured with a rotarod test, which suggested that PD animals did not show an obvious reduction in time on the rod at one, two, and three weeks after the first MPTP injection. However, over time, a significant decrease was obtained. Additionally, a progressive decline was observed between four and five weeks. These results indicated that motor impairment could only be obtained four weeks after the MPTP injection, with a progressive impairment during four to five weeks (Fig. 1a) (*n* = 8). To further investigate the change in the dopaminergic neurons of basal ganglia, the tyrosine hydroxylase (TH) levels in the striatum and SN were evaluated. We found that TH levels were significantly decreased at four, five, and six weeks after the injection, with a progressive decline between the four and five-week time points (Fig. 1b–d) (*n* = 6). The TH^+^ neuron and neurites in the SN and striatum were similar with the above results (Fig. 1e–g) (*n* = 6). Therefore, we performed STN-DBS four weeks after the first MPTP injection to mimic the clinical course of PD, with a stimulation duration of one week.

### The accurate location of STN-DBS

**Fig. 2:**
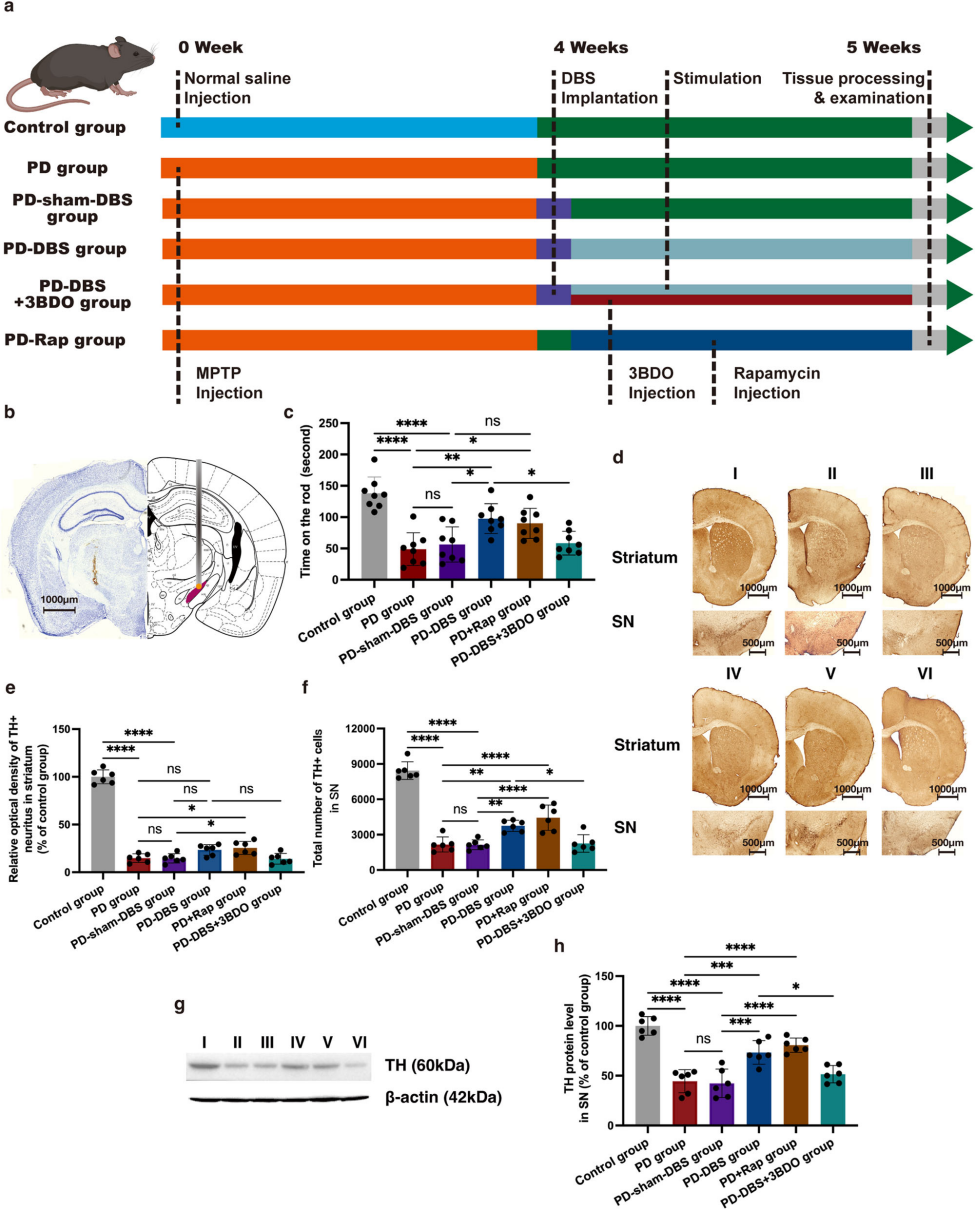
The neuroprotection of STN-DBS in a PD mouse model. **a** Experimental design of the mouse STN-DBS. **b** Nissl staining of lead implantation. The lead accurately targeted the STN region. Red area indicates the STN region of the mouse brain in the atlas^70^. **c** The rotarod test of different groups. STN-DBS and rapamycin extended the time on the rod of the PD mouse model; however, 3BDO inhibited this effect (*n* = 8 per group; F_(5,42)_ = 15.08, *P* < 0.0001; one-way ANOVA followed by a Tukey post-hoc correction). **d** Immunohistochemistry staining of TH in different groups. **e**, **f** STN-DBS, as well as rapamycin, relieved the tendency of reduction of TH^+^ cells in SN. Only rapamycin significantly elevated the TH^+^ neurites in the striatum, and STN-DBS merely obtained a tendency to increase (*n* = 6 per group; TH^+^ neurons: F_(5,30)_ = 197.3, *P* < 0.0001; TH^+^ neurites: F_(5,30)_ = 69^.^51, *P* < 0.0001; one-way ANOVA followed by a Tukey post-hoc correction). **g** Western blot analysis of TH in the SN of different groups. **h** PD-DBS and PD+Rap groups showed an increase in TH level in the SN by comparison with PD and PD-sham-DBS groups; however, 3BDO obstructed the preservation of TH by STN-DBS (*n* = 6 per group; F_(5,30)_ = 27.7, *P* < 0.0001; one-way ANOVA followed by a Tukey post-hoc correction). I: control group; II: PD group; III: PD-sham-DBS group; IV: PD-DBS group; V: PD+Rap group; VI: PD-DBS + 3BDO group. ^*^P < 0.05; ^**^P < 0.01; ^***^P < 0.001; ^****^P < 0.0001. Error bars: standard deviation of the mean. STN-DBS subthalamic nuclei deep brain stimulation, PD Parkinson’s disease, SN substantia nigra, TH tyrosine hydroxylase, MPTP 1-methyl-4-phenyl-1,2,3,6-tetrahydropyridine, ns not significant.

The lead location in mice was developed by using Nissl staining. The results indicated that the lead correctly targeted the STN region in all mice, and these animals were used in the subsequent experiments (Thirteen mice were removed from the experiments, due to misplacing electrodes.) (Fig. 2a, b).

### STN-DBS relieved motor impairment and loss of dopaminergic neurons in the PD model

As mentioned above, the MPTP rodent PD model exhibited a motor impairment. We found that STN-DBS could extend the time spent on the rods in the PD mice model (Fig. 2c) (*n* = 8).

We assessed dopaminergic neuron loss in different groups to investigate whether the reduction in motor performance was attributed to a change in dopaminergic neuron loss. The PD and PD-sham-DBS groups showed a severe reduction in TH^+^ cell number, but STN-DBS relieved this tendency. Nevertheless, TH^+^ neurites in the striatum were only increased slightly, without a significant *P* value (*P* = 0.1430, vs PD group; *P* = 0.1263, vs PD-sham-DBS group) (Fig. 2d–f) (*n* = 6). Additionally, the TH levels in SN were evaluated via western blotting, the PD-DBS group showed an increase in TH level in SN by comparison with PD and the PD-sham-DBS group (Fig. 2g, h) (*n* = 6). These results indicate that STN-DBS could reduce dopaminergic neuron loss in SN in the PD rodent model.

### The neuroprotective effect of STN-DBS is mediated by mTOR-dependent mitophagy

**Fig. 3:**
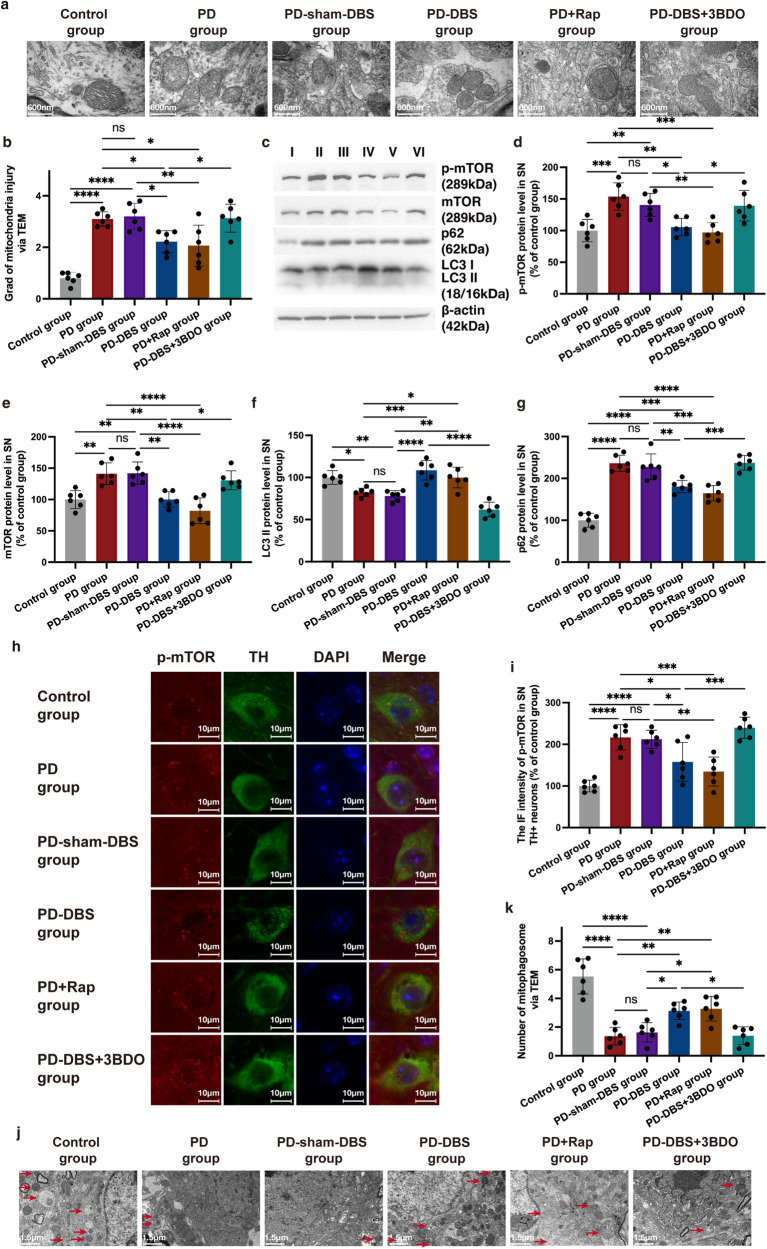
STN-DBS contributed a neuroprotective effect in PD mice through mTOR-dependent mitophagy. **a** The integrity of mitochondria (neuron) measured via TEM. Magnification: 60,000×. **b** Marked mitochondrial ultrastructural injury was observed in the PD and PD-sham-DBS groups. In contrast, mitochondria in the PD-DBS and PD+Rap groups showed less injury, which disappeared after treatment with 3BDO (*n* = 6 per group; F_(5,30)_ = 20.9, *P* < 0.0001; one-way ANOVA followed by a Tukey post-hoc correction). **c** Western blot analysis of p-mTOR, mTOR, LC3, and p62 in different groups (shared with the same mice in Fig. 2g). **d**, **e** The mTOR and p-mTOR expressions were suppressed by STN-DBS in the PD mouse model, and this phenomenon disappeared when intervened with 3BDO (*n* = 6 per group; mTOR: F_(5,30)_ = 14.1, *P* < 0.0001; p-mTOR: F_(5,30)_ = 10.3, *P* < 0.0001; one-way ANOVA followed by a Tukey post-hoc correction). **f** STN-DBS reversed the decrease in LC3 II, and a similar result was also obtained in PD mice treated with rapamycin (F_(5,30)_ = 22.3, *P* < 0.0001; one-way ANOVA followed by a Tukey post-hoc correction). **g** The increased p62 expression in PD models was reversed by STN stimulation, which was inhibited by 3BDO injection (*n* = 6 per group; F_(5,30)_ = 42.9, *P* < 0.0001; one-way ANOVA followed by a Tukey post-hoc correction). **h** Co-localization of TH and p-mTOR via IF staining. **i** The p-mTOR expression was elevated in the dopaminergic neurons of PD but was decreased by STN-DBS and rapamycin. With the injection of 3BDO, the p-mTOR expression in dopaminergic neurons was not further influenced by STN-DBS (*n* = 6 per group; F_(5,30)_ = 19.1, *P* < 0.0001; one-way ANOVA followed by a Tukey post-hoc correction). **j** Mitophagosomes (neuron) observed via TEM in different groups (shared with the same mice in Fig. 3a). Magnification: 20,000×. **k** Fewer mitophagosomes (white arrows) were observed in the PD mice compared with the control. However, STN-DBS and rapamycin induced a marked increase in the number of mitophagosomes in the SN of the PD model (*n* = 6 per group; F_(5,30)_ = 24.8, *P* < 0.0001; one-way ANOVA followed by a Tukey post-hoc correction). I: control group; II: PD group; III: PD-sham-DBS group; IV: PD-DBS group; V: PD+Rap group; VI: PD-DBS + 3BDO group. ^*^P < 0.05; ^**^P < 0.01; ^***^P < 0.001; ^****^P < 0.0001. Error bars: standard deviation of the mean. STN-DBS subthalamic nuclei deep brain stimulation, PD Parkinson’s disease, SN substantia nigra, IF immunofluorescence, DAPI 4′,6-diamidino-2-phenylindole, TH tyrosine hydroxylase, p-mTOR phospho-mTOR, TEM transmission electron microscopy, ns not significant.

Mitochondrial failure and reduced mitophagy have been proposed as important components in determining pathological heterogeneity and selective vulnerability of PD^17^. The integrity of mitochondria was directly measured via a transmission electron microscope (TEM). A marked mitochondrial ultrastructural injury was observed in the PD and PD-sham-DBS groups, with significant swelling of the mitochondrial matrix. In some instances, mitochondrial swelling was accompanied by the disruption of membrane integrity. In contrast, mitochondria in the PD-DBS group had relative normal morphology, with slight evidence of swelling, outer membrane breakage, or intracristal dilation, which was closer to that of the control group (Fig. 3a, b) (*n* = 6).

The predominant effect of mTOR activation is to suppress mitophagy^16^. We found that phospho-mTOR (p-mTOR, Ser2448) and mTOR levels were both elevated in PD and PD-sham-DBS groups due to the MPTP injection. Rapamycin, also known as sirolimus, forms a complex with FK506-binding protein 12 and in this form inhibits the activity of mTOR^12^. In the present study, rapamycin could successfully decline the p-mTOR and mTOR expression in the PD model. Interestingly, the mTOR pathway was suppressed by STN-DBS in the PD mouse model, and this phenomenon disappeared when intervened with 3BDO (mTOR activator) (Fig. 3c–e) (*n* = 6). The results above suggested STN stimulation regulated the activation of the mTOR pathway in the PD mice, however, different types of cells exist in SN, and whether it involved dopaminergic neurons still needs to be further measured. Therefore, immunofluorescence (IF) staining was conducted to study the colocalization of p-mTOR and the dopaminergic neuron marker. It was found that p-mTOR expression was elevated in the dopaminergic neuron of PD, but decreased by STN-DBS, compared with the model without stimulation. With the injection of 3BDO, the mTOR activation in dopaminergic neurons was not further influenced by STN-DBS (Fig. 3h, i) (*n* = 6).

Similar to the mice treated with STN-DBS, mice that received an injection of rapamycin also showed a better performance in the rotarod test **(**Fig. 2c) (*n* = 8), accompanied by preserved dopaminergic neurons (Fig. 2d–h) (*n* = 6). The therapeutic efficacy of STN-DBS on motor symptoms and neuroprotection of dopaminergic neurons dispersed when the mTOR activator was applied. One PD mice group was treated with both STN-DBS and 3BDO-mTOR activator and did not show any improvement in motor performance or preserved dopaminergic neurons, indicating a blocking effect on the STN-DBS (Fig. 2c, d–h) (*n* = 8; *n* = 6).

The expressions of LC3 II, an autophagosome marker reflecting autophagy activity^12,19^, were reduced in the SN of the PD and PD-sham-DBS groups, whereas STN-DBS was found to reverse the tendency, a similar result was obtained in PD mice treated with rapamycin (Fig. 3c, f). SQSTM1/p62, an autophagic adapter, is recruited to mitochondrial clusters and is essential for the clearance of mitochondria^21^. The increased p62 expression in PD models was reversed by STN stimulation, which was inhibited in the PD-DBS + 3BDO group (Fig. 3c, g) (*n* = 6). Mitophagy, the selective autophagy of mitochondria, needed to be further evaluated by IF and TEM. Significantly, the STN stimulation was able to increase the co-localization of TOMM20 (mitochondria marker) and LC3 in the TH^+^ cells in the SN of the PD model, nevertheless, this could be interrupted by treating with the mTOR activator (Supplementary Fig. 1a, b) (*n* = 6). Additionally, the number of mitophagosomes was also measured by TEM. Fewer mitophagosomes were observed in the PD and PD-sham-DBS groups compared with the control group. Nevertheless, STN-DBS and rapamycin induced a marked increase in the number of mitophagosomes (Fig. 3j, k) (*n* = 6). These results indicated that STN-DBS could exert an improvement in the mitophagy of dopaminergic neurons via an mTOR-dependent pathway.

### STN-DBS alleviates oxidative stress and mitochondrial dysfunction via mitophagy

**Fig. 4:**
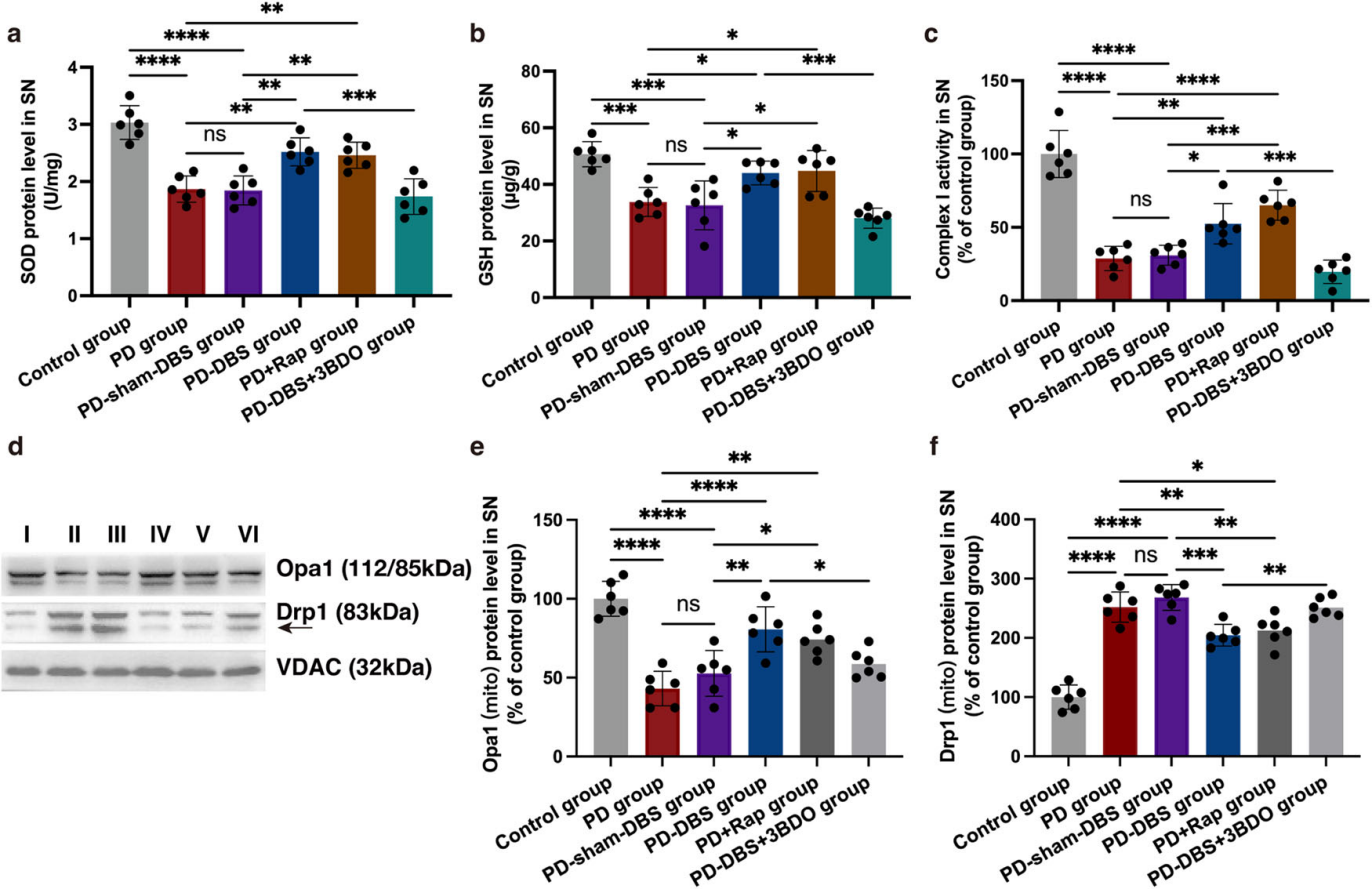
STN-DBS alleviates oxidative stress and stabilizes mitochondrial homeostasis. **a**, **b** SOD and GSH levels in SN measured via ELISAs. An increased expression of SOD and GSH in SN, compared with the mice that received no stimulation or rapamycin (only SOD) injection. The mTOR activator 3BDO inhibited this effect (*n* = 6 per group; SOD: F_(5,30)_ = 22.6, *P* < 0.0001; GSH: F_(5,30)_ = 13.6, *P* < 0.0001; one-way ANOVA followed by a Tukey post-hoc correction). **c** Complex I activity in SN measured via ELISAs. Complex I had higher activity in PD mice that received STN stimulation or rapamycin treatment, however, the mTOR activator could suppress the effect of STN stimulation on the activity of complex I (*n* = 6 per group; F_(5,30)_ = 44.0, *P* < 0.0001; one-way ANOVA followed by a Tukey post-hoc correction). **d** Western blot analysis of Drp1 and Opa1 in mitochondria of SN. **e**, **f** Opa1 expression in mitochondria was decreased in PD mice, whereas, an elevated expression of Opa1 was detected in PD mice treated with STN stimulation (*n* = 6 per group; F_(3,20)_ = 26.3, *P* < 0.0001; one-way ANOVA followed by a Tukey post-hoc correction). On the contrary, the opposite trend of Drp1 expression was observed in the mitochondria of SN (*n* = 6 per group; F_(3,20)_ = 74.6, *P* < 0.0001; one-way ANOVA followed by a Tukey post-hoc correction). I: control group; II: PD group; III: PD-sham-DBS group; IV: PD-DBS group; V: PD+Rap group; VI: PD-DBS + 3BDO group. ^*^P < 0.05; ^**^P < 0.01; ^***^P < 0.001; ^****^P < 0.0001. Error bars: standard deviation of the mean. STN-DBS subthalamic nuclei deep brain stimulation, PD Parkinson’s disease, SN substantia nigra, ELISA enzyme-linked immunosorbent assay, SOD superoxide dismutase, GSH glutathione, ns not significant.

Mitophagy is essential for the elimination of damaged mitochondria and alleviates oxidative stress^16^. The antioxidant enzyme levels were measured to reflect the level of oxidative stress. Both SOD and glutathione (GSH) were down-regulated in the SN of PD mice, whereas, rapamycin was able to rescue the decreased expression of these antioxidant enzymes. Meanwhile, STN-DBS exerted a ‘rapamycin’ like effect, increasing the expression of SOD and GSH in SN, compared with the mice without stimulation, indicating that STN-DBS played an antioxidant role in PD mice, which was disrupted by using an mTOR activator (Fig. 4a, b) (*n* = 6).

Complex I activity can reflect mitochondrial function. Complex I was detected with increased activity in PD mice that had received STN stimulation. Nevertheless, treating the mice with an mTOR activator could suppress the activity of complex I (Fig. 4c) (*n* = 6).

### STN-DBS regulates mitochondrial homeostasis

Mitochondrial homeostasis is maintained by mitochondrial fission and fusion, which is catalyzed by the Drp1 and Opa1, respectively^22^. Opa1 protein expression in mitochondria was decreased in the PD and PD-sham-DBS groups, whereas an elevated expression was detected in PD mice treated with STN stimulation, and this effect could be interrupted by 3BDO injection. However, the opposite trend in Drp1 protein expression was observed in SN mitochondria (Fig. 4d–f) (*n* = 6). These observations highlight how STN-DBS stabilizes mitochondrial homeostasis via up-regulating mitochondrial fusion and down-regulating mitochondrial fission.

### Mitochondria-mediated apoptosis is suppressed by STN-DBS

**Fig. 5:**
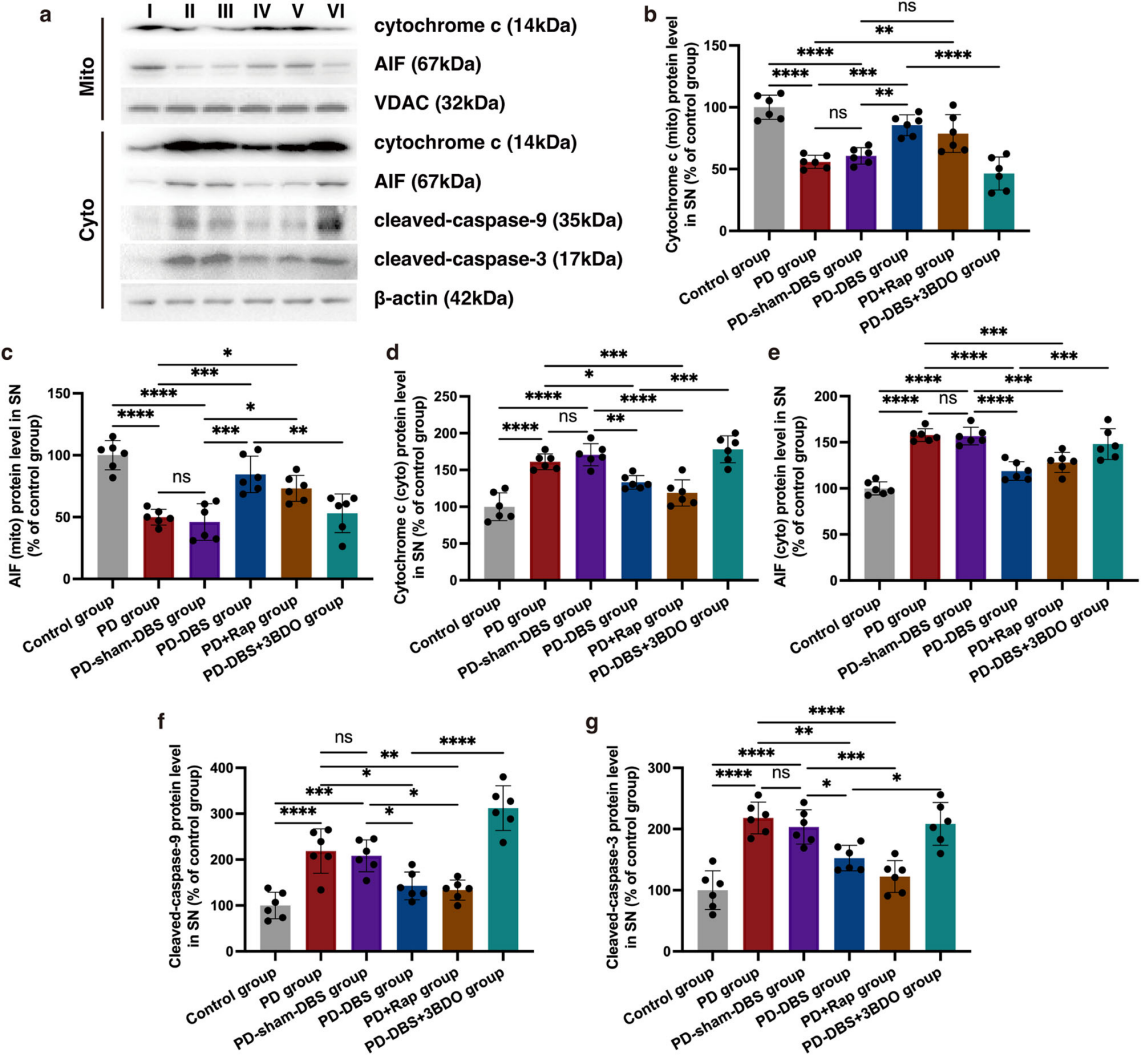
Mitochondrial-mediated apoptosis is suppressed by STN-DBS. **a** Western blot analysis of cytochrome c, AIF, cleaved-caspase-3 and -9 in the cytoplasm and mitochondria of SN. **b**–**e** In the cytoplasm, significantly more AIF was detected in PD mice; however, that was reduced by STN-DBS and rapamycin injection. The efficacy of STN stimulation on AIF expression in the cytoplasm was blocked by 3BDO (*n* = 6 per group; F_(5,30)_ = 28.2, *P* < 0.0001; one-way ANOVA followed by a Tukey post-hoc correction). The tendency of AIF expression in mitochondria among the different groups was opposite to that in the cytoplasm (*n* = 6 per group; F_(5,30)_ = 17.5, *P* < 0.0001; one-way ANOVA followed by a Tukey post-hoc correction). Moreover, cytochrome c showed a similar expression tendency both in cytoplasm (*n* = 6 per group; F_(5,30)_ = 24.4, *P* < 0.0001; one-way ANOVA followed by a Tukey post-hoc correction) and mitochondria (*n* = 6 per group; F_(5,30)_ = 22.6, *P* < 0.0001; one-way ANOVA followed by a Tukey post-hoc correction). **f**, **g** The cleaved-caspase-9 (F_(5,30)_ = 26.1, *P* < 0.0001) and -3 (F_(5,30)_ = 18.5, *P* < 0.0001) were up-regulated in PD mice, on the contrary, they were suppressed by STN stimulation and rapamycin treatment (*n* = 6 per group; one-way ANOVA followed by a Tukey post-hoc correction). I: control group; II: PD group; III: PD-sham-DBS group; IV: PD-DBS group; V: PD+Rap group; VI: PD-DBS + 3BDO group. ^*^P < 0.05; ^**^P < 0.01; ^***^P < 0.001; ^****^P < 0.0001. Error bars: standard deviation of the mean. STN-DBS subthalamic nuclei deep brain stimulation, PD Parkinson’s disease, SN substantia nigra, AIF apoptosis-inducing factor, ns not significant.

As mentioned above, TEM was used to evaluate mitochondrial injury. PD mice injected with rapamycin showed an alleviation in mitochondrial injury, which was similar to in PD mice that received STN stimulation. Serious injury was obtained in the PD-DBS + 3BDO group. This phenomenon was scored and confirmed by the criteria of mitochondrial injury (Fig. 3a, b) (*n* = 6). Mitochondria-related apoptogenic factors, including apoptosis-inducing factor (AIF) and cytochrome c, can leak from damaged mitochondria^23^. In the cytoplasm, significantly more AIF was detected in PD mice, however, that was reduced by STN stimulation or rapamycin injection. The efficacy of STN stimulation on AIF detection in the cytoplasm was inhibited by 3BDO. The tendency of AIF expression in mitochondria among the different groups was opposite to that in the cytoplasm. Moreover, cytochrome c showed a similar trend in expression in both cytoplasm and mitochondria (Fig. 5a–e) (*n* = 6). Our results suggest that STN-DBS could reduce the leakage of AIF and cytochrome c from the mitochondria to the cytoplasm, through an mTOR-dependent pathway. Cytochrome c release induces caspase activation, which in turn promotes cell apoptosis. Cleaved-caspase-9 and -3 were up-regulated in PD mice, which leads to dopaminergic neuron loss. STN stimulation and rapamycin could suppress apoptosis-associated caspase activation, which could be inhibited by an mTOR activator (Fig. 5a, f, g) (*n* = 6).

### Chronic STN-DBS exerts an “antioxidant” role and stabilizes mitochondrial homeostasis in the PD monkey model

**Fig. 6:**
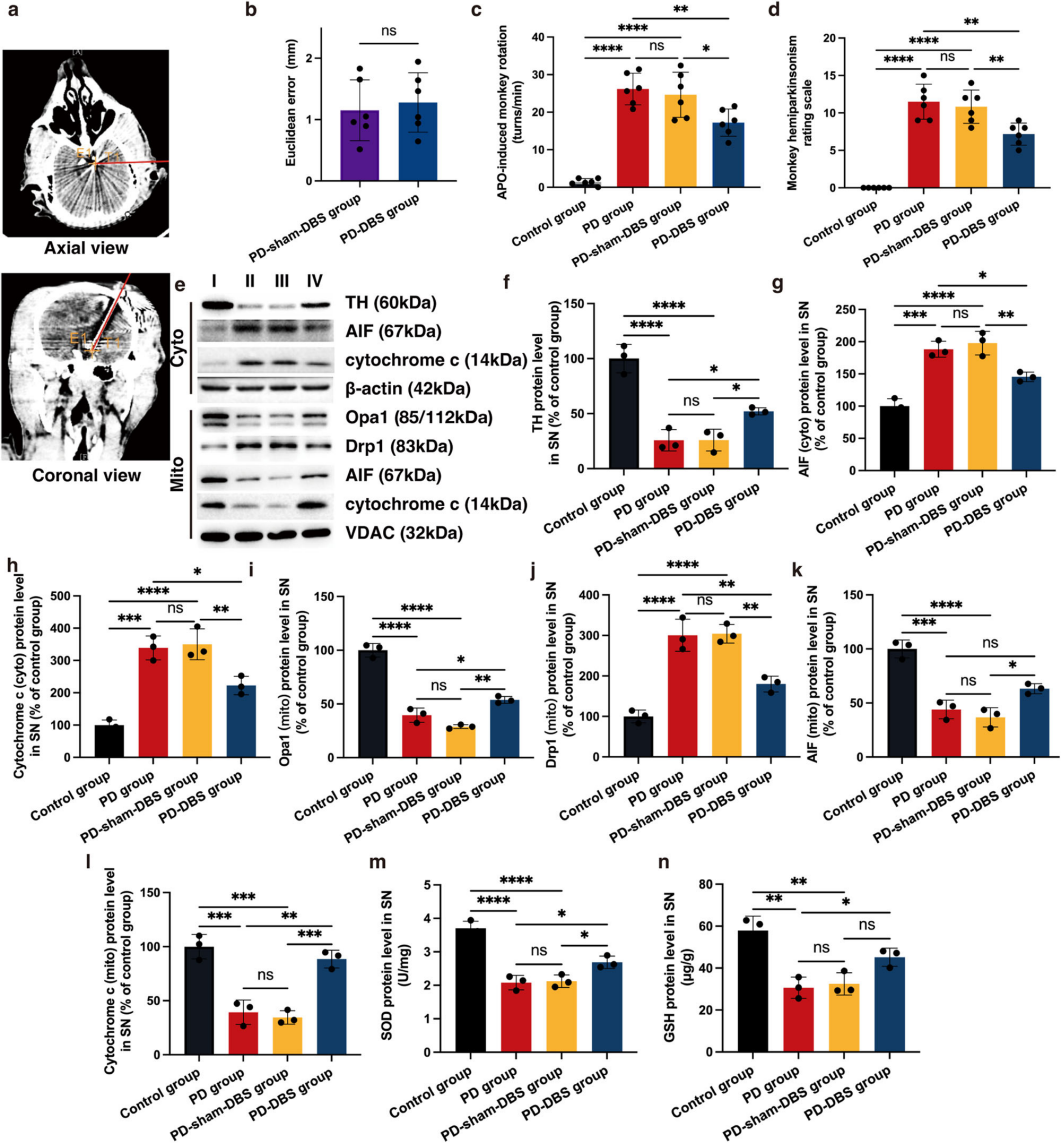
Chronic STN-DBS exerts an “antioxidant” role and stabilizes mitochondrial homeostasis in a PD monkey model. **a** The position of the DBS lead in postoperative CT (merged with preoperative surgical planning). It was observed that the actual position of the DBS lead was approximately overlayed by the surgical planning, suggesting that the DBS lead accurately targeted the STN region. The red line indicates the trajectory of surgical planning. **b** Euclidean error in different groups. There was no significant difference in Euclidean error between PD-sham-DBS and PD-DBS groups, and all lead positions showed an ideal accuracy (*n* = 6 per group; *P* = 0.9632; two-sample t-test). **c** APO-induced rotation in different groups. After STN-DBS for two months, monkeys showed a significantly reduced cyclomatic number (*n* = 6 per group; F_(3,20)_ = 45.2, *P* < 0.0001; one-way ANOVA followed by a Tukey post-hoc correction). **d** The hemiparkinsonism rating score in different groups. After the establishment of the PD model with MPTP, monkeys showed fixed posture, reduced arm movements, and elevated scores based on the hemiparkinsonism rating scale. After STN stimulation, the symptoms were relieved (*n* = 6 per group; F_(3,20)_ = 5.8, *P* = 0.0051; one-way ANOVA followed by a Tukey post-hoc correction). **e** Western blot analysis of TH, cytochrome c, AIF, Drp1, and Opa1 in the cytoplasm and mitochondria of SN. **f** TH expression was down-regulated in PD and PD-sham-DBS groups, however, it was up-regulated by STN stimulation (*n* = 3 per group; F_(3,8)_ = 39.6, *P* < 0.0001; one-way ANOVA followed by a Tukey post-hoc correction). **g**, **h** More AIF (mito: F_(3,8)_ = 39.4, *P* < 0.0001; cyto: F_(3,8)_ = 35.6, *P* < 0.0001) and cytochrome c (mito: F_(3,8)_ = 37.4, *P* < 0.0001; cyto: F_(3,8)_ = 34.7, *P* < 0.0001) were detected in the mitochondria after STN stimulation (*n* = 3 per group; one-way ANOVA followed by a Tukey post-hoc correction). **i**, **j** Opa1 expression in mitochondria was increased by STN-DBS (*n* = 3 per group; F_(3,8)_ = 121.4, *P* < 0.0001; one-way ANOVA followed by a Tukey post-hoc correction); however, Drp1 was down-regulated by this treatment (*n* = 3 per group; F_(3,8)_ = 43.3, *P* < 0.0001; one-way ANOVA followed by a Tukey post-hoc correction). **k**, **l** Monkeys in the PD-DBS group were observed to have lower expression of AIF (mito: F_(3,8)_ = 39.4, *P* < 0.0001; cyto: F_(3,8)_ = 35.6, *P* < 0.0001) and cytochrome c (mito: F_(3,8)_ = 37.4, *P* < 0.0001; cyto: F_(3,8)_ = 34.7, *P* < 0.0001) in the cytoplasm, compared with that of PD (a tendency of AIF, *P* = 0.0650) and PD-sham-DBS group (*n* = 3 per group; one-way ANOVA followed by a Tukey post-hoc correction). **m**, **n** SOD (F_(3,8)_ = 43.4, *P* < 0.0001) and GSH (F_(3,8)_ = 16.1, *P* = 0.0009) levels in SN were significantly increased by chronic STN stimulation compared with PD monkeys without treatment (*n* = 3 per group; one-way ANOVA followed by a Tukey post-hoc correction). I: control group; II: PD group; III: PD-sham-DBS group; IV: PD-DBS group; V: PD+Rap group; VI: PD-DBS + 3BDO group. ^*^P < 0.05; ^**^P < 0.01; ^***^P < 0.001; ^****^P < 0.0001. Error bars standard deviation of the mean, STN-DBS subthalamic nuclei deep brain stimulation, PD Parkinson’s disease, SN substantia nigra, TH tyrosine hydroxylase, MRI magnetic resonance imaging, CT computed tomography, APO apomorphine, AIF apoptosis-inducing factor, ELISA enzyme-linked immunosorbent assay, SOD superoxide dismutase, GSH glutathione, ns not significant.

Besides the differences in basal ganglia structure between the NHP and rodent, whether a much longer stimulation could exert a neuroprotective role via the anti-oxidative stress still needs to be explored. Hence, we developed a monkey experiment (Supplementary Fig. 2a, b). The accuracy of neurosurgical robot-assisted DBS implantation has been confirmed by our previous study^24^. By comparison with the surgical plan via image fusion, the Euclidean errors were 1.15 ± 0.50 and 1.28 ± 0.49 mm in the PD-sham-DBS and PD-DBS groups, respectively, suggesting no obvious difference (Fig. 6a, b) (*n* = 6). Apomorphine (APO)-induced rotation was applied to test motor impairment. Single-side rotation was observed at a high speed in the PD model, after APO injection. After treatments with STN-DBS for two months, monkeys showed a significantly reduced cyclomatic number (Fig. 6c) (*n* = 6). Additionally, a zero score was obtained in control animals on the hemiparkinsonism rating scale because no symptoms were observed. The monkeys in the PD and PD-sham-DBS groups achieved a relatively high score, with symptoms including fixed posture, reduced arm movements, etc. However, monkeys that received STN stimulation appeared to have fewer tremors and more arm movement, which resulted in an obvious decrease in the score (Fig. 6d) (*n* = 6). Moreover, we tested whether the reduced cyclomatic number and hemiparkinsonism rating score contributed to the neuroprotection of STN-DBS, and observed that TH expression was up-regulated by STN stimulation, which indicated a dopaminergic neuroprotection of long-term STN stimulation in the PD monkey (Fig. 6e, f) (*n* = 3).

Besides the behavior tests, we evaluated the mechanism at the molecular level. The levels of antioxidant enzymes were measured. We found that the SOD expression in SN was significantly increased by chronic STN stimulation compared with the PD monkey (Fig. 6m) (*n* = 3). Similarly, GSH was down-regulated in SN of PD and PD-sham-DBS groups and was reversed by STN-DBS (a significant increase vs PD group; an elevated tendency vs the PD-sham-DBS group, *P* = 0.0835) (Fig. 6n) (*n* = 3). In another aspect, we evaluated the mitochondrial homeostasis of SN. Similar to the mouse experiment, Opa1 expression in mitochondria was increased by STN-DBS. Meanwhile, Drp1 was down-regulated by this treatment, indicating that mitochondrial homeostasis was maintained by long-term STN stimulation (Fig. 6e, i, g). Less AIF and cytochrome c were detected in the cytoplasm of monkeys receiving STN stimulation, with a contrary tendency in mitochondria, which indicated that a lower level of these apoptogenic factors was released from mitochondria (Fig. 6e, g, h, k, l). Most of these results were similar to those of the mice experiments, which suggests that long-term STN stimulation could alleviate oxidative stress and stabilize mitochondrial homeostasis, and plays a neuroprotective role in NHP.

### STN-DBS suppressed oxidative stress in PD patients

Although the mechanism of STN-DBS has been confirmed not only in rodents but also NHP, whether the antioxidative stress effects could be observed in the clinic is still unknown. Therefore, we recruited PD patients with STN-DBS and collected cerebrospinal fluid (CSF) pre- and post-operation.

**Fig. 7:**
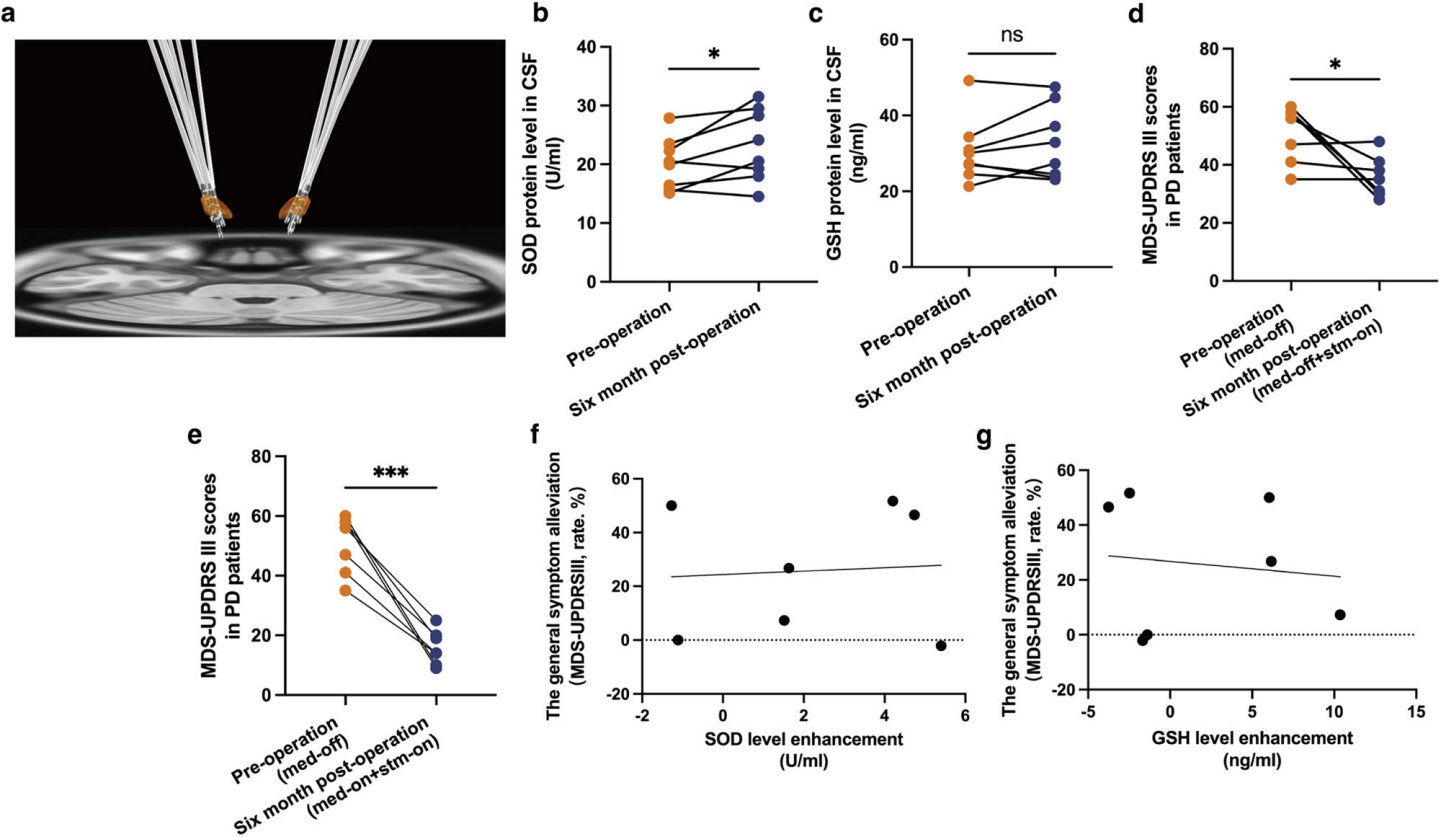
STN-DBS suppressed oxidative stress in PD patients. **a** DBS lead construction via LeadDBS toolbox. Bilateral DBS leads accurately targeted the STN region (orange area). **b**, **c** The SOD level was obviously increased after patients received STN-DBS for six months (*P* = 0.0455), nevertheless, GSH expression did not change in the same period (*P* = 0.3025) (*n* = 8 per time point; paired-T test). **d**, **e** Regardless of whether patients were on or off medication, the MDS-UPDRS III scores were significantly reduced after the operation (*n* = 7 per time point; d: *P* = 0.0329; e: *P* = 0.0002; paired-T test). **f**, **g** There was no significant relationship between changes in antioxidant enzymes [SOD (*P* = 0.8763) or GSH (*P* = 0.7926)] and change in MDS-UPDRS III ($\frac{{({Preoperative}\,{\rm{MDS}}-{\rm{UPDRS}})}_{{med}-{off}}-{({Postoperative}\,{MDS}\,{UPDRS})}_{{stm}-{on},\,{med}-{off}}}{{({Preoperative}\,{\rm{MDS}}-{\rm{UPDRS}})}_{{med}-{off}}}\times 100 \%$) (*n* = 7; Pearson correlation test). ^*^P < 0.05; ^**^P < 0.01; ^***^P < 0.001; ^****^P < 0.0001. Error bars standard deviation of the mean, STN-DBS subthalamic nuclei deep brain stimulation, PD Parkinson’s disease, SOD superoxide dismutase, GSH glutathione, MDS-UPDRS Movement Disorder Society-Sponsored Revision of the Unified Parkinson’s Disease Rating Scale, ns not significant.

The lead positions in PD patients were confirmed with a MATLAB toolbox-LeadDBS (v2.1.8, https://www.lead-dbs.org) via fusion of preoperative magnetic resonance imaging (MRI) and postoperative computed tomography (CT)^25^, the results showed that the leads were accurately implanted into the STN (Fig. 7a). We compared the SOD and GSH pre-operation and six months post-operation. The SOD level was obviously increased after patients received the treatment, nevertheless, GSH expression did not seem to change during the same period (Fig. 7b, c) (*n* = 8). We were curious whether changes in oxidative stress were correlated with the alleviation of symptoms. In this cohort of patients, the Movement Disorder Society-Sponsored Revision of the Unified Parkinson’s Disease Rating Scale (MDS-UPDRS III) scores were significantly reduced after the operation, suggesting a therapeutic efficacy on motor symptoms (Fig. 7d, e) (*n* = 7). However, we did not observe a significant relationship between the change in antioxidant enzymes (SOD or GSH) and MDS-UPDRS III scores (all *Ps* > 0.05) (Fig. 7f, g) (*n* = 7). Moreover, we divided MDS-UPDRS III into several dimensions, to score the different symptoms of PD, according to the previous study^26^. Still, no obvious correlation was observed between the change in levels of SOD or GSH, and alteration in tremors, rigidity, bradykinesia, and axial symptoms (all *Ps* > 0.05) (Supplementary Fig 3a–h) (*n* = 7).

## Discussion

**Fig. 8:**
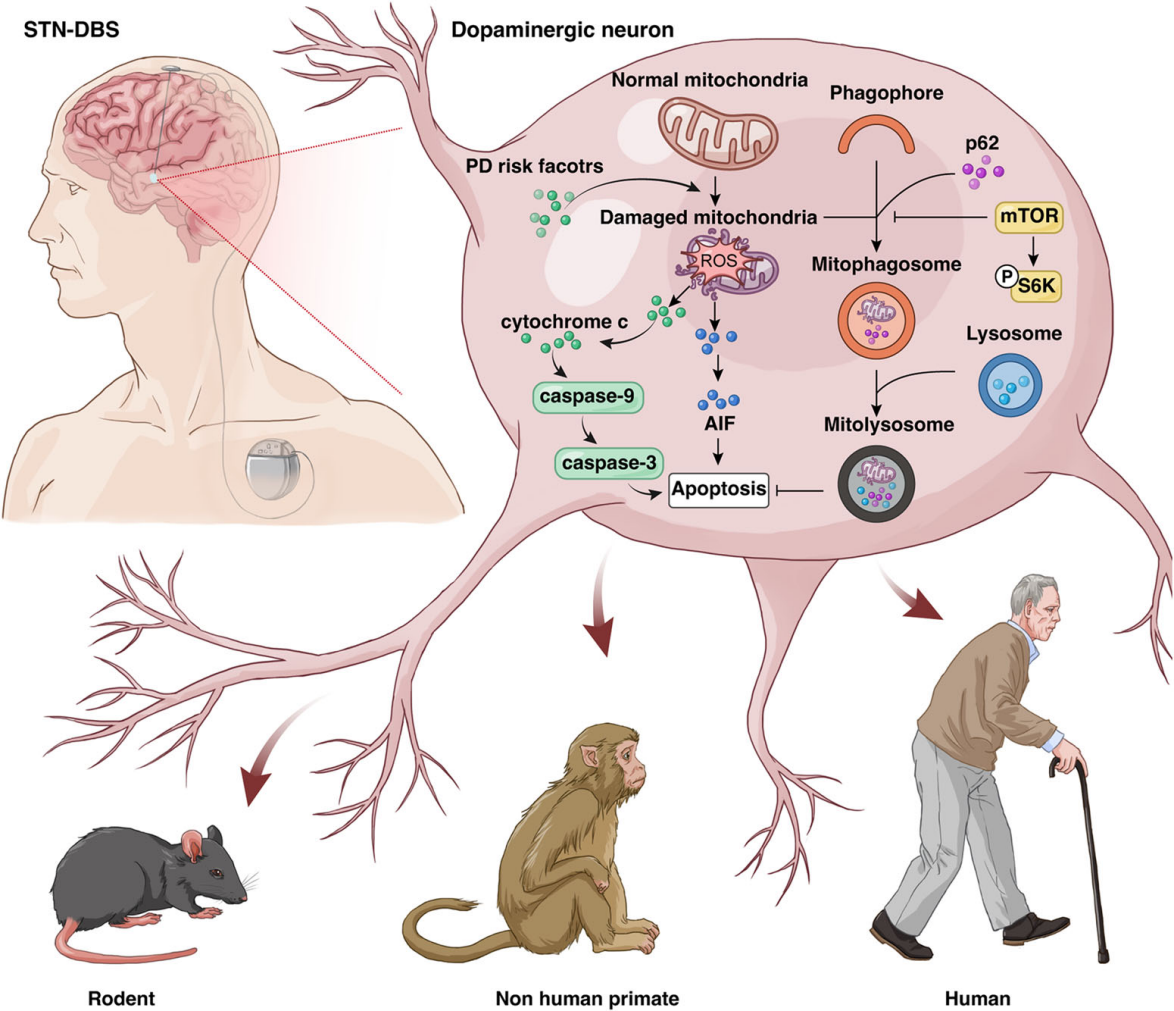
Schematic illustration of the neuroprotective effects of STN-DBS. STN-DBS was able to increase the mitophagy in dopaminergic neurons via an mTOR-dependent pathway, and the oxidative stress was suppressed due to removal of damaged mitochondria. As a consequence, the apoptogenic factors released from mitochondria were reduced, which was finally attributed to the dopaminergic neuroprotection of STN-DBS in the SN of PD. STN-DBS subthalamic nuclei deep brain stimulation, PD Parkinson’s disease, SN substantia nigra, AIF apoptosis-inducing factor.

STN-DBS is commonly indicated for PD and became the standard of care for this neurodegenerative disease^27^. However, the mechanism of this neuromodulation technique at a molecular level still needs to be further illustrated. Here, we showed that STN-DBS could play an “antioxidant” role in PD, which is dependent on an increase in mitophagy via an mTOR pathway. The “antioxidant” effect of STN-DBS reduced the apoptosis of dopaminergic neurons and exerted a neuroprotection effect, which inhibited disease progression (Fig. 8).

These findings were first confirmed in the rodent experiment with a short period of stimulation. However, the results from a rodent experiment only were not convincing enough. Several important structures in the basal ganglia are implicated in specific neural circuits and the disease progression of PD^28,29^, and these structures have major differences between NHP and rodents. For instance, in rodents, the dorsal striatum is named neostriatum and it could be divided into a dorsomedial and a dorsolateral part, while in monkeys and humans it is divided into the caudate nucleus and putamen. In parallel, both in humans and the NHP, the internal and external segments of the globus pallidus are structurally divided by the internal lamina and are placed close to each other. Rodents lack such structural separation^30^. Additionally, only short-term stimulation was delivered in the rodent experiment, whether a long-term stimulation could exert neuroprotection, and through a similar mechanism remains unknown. The previous study also addressed the limitation of rodents in the DBS study^31^. Therefore, we illustrated these issues and confirmed our findings, using the tissue from NHP and CSF from patients after long-term stimulation.

Even though the majority of STN fibers terminate at the level of the reticulate part, a few fibers ascend along the dopaminergic cell columns of the SN pars compacta (SNc), thereby exerting an influence on both dopaminergic and non-dopaminergic cells^32^. Cortical information received at the input stations is transmitted to the output nuclei via three distinct pathways: (1) Direct pathway: striatal neurons expressing substance P receive cortical inputs and project directly to the internal globus pallidus (GPi)/ SN pars reticulata (SNr). (2) Indirect pathway: striatal neurons expressing enkephalin receive cortical inputs and transmit to the GPi/SNr via a polysynaptic route involving the external globus pallidus (GPe) and STN. (3) Hyperdirect pathway: STN neurons receive direct cortical inputs and project directly to the GPi/SNr^33^. Therefore, the function of SN may be influenced via modifying STN.

DBS was approved by the Food and Drug Administration in 2002 as one of the therapies for PD. Randomized controlled clinical trials of STN-DBS have been performed and showed a marked reduction in motor severity and an increase in the quality of daily life^34^. DBS can obtain an immediate effect on the firing rate and pattern of individual neurons and neurotransmitters in the basal ganglia^35^. The major unmet need in the management of PD is to slow down the progression of the disease and reduce or prevent key disability milestones, however, no medication has been confirmed to inhibit the progression. Fortunately, the studies involve patients with an early stage of PD treated with STN-DBS and long-term follow-up could give important insights into the neuroprotective potential of DBS^7,36,37^. Moreover, in observations via neuroimaging in PD patients, STN-DBS showed a specific pattern of changes in the motor circuit, including increased ligand uptake in the basal ganglia^38^. Our previous study showed rats treated with STN-DBS increased the survival of dopaminergic neurons in the SN^19^. Several studies including our own tried to investigate how STN-DBS exerts a neuroprotective effect. There is an interesting issue that only high-frequency stimulation delivered to STN could increase the fractional anisotropy value of the SN and exert a neuroprotective effect^39^. Fischer et al. found that STN-DBS activates BDNF-trkB signaling, accompanied by phosphorylation of Akt in SN neurons^40^. Moreover, STN stimulation has been found to protect against α-synuclein induced dopaminergic neuron loss^8^. Our former findings indicated that the neuroprotective effect was attributed to activated autophagy^19^. Another study of ours showed a reduction of inflammatory mediators in microglia via the modulation of CX3CL1/CX3CR1 and ERK signaling, finally alleviates the apoptosis of SN neurons^41^.

Some studies found neuroprotection was achieved via influencing mitophagy. For instance, celastrol has been confirmed to exerts neuroprotection in PD by activating mitophagy to degrade impaired mitochondria and further inhibit dopaminergic neuronal apoptosis^42^, and Artemisia Leaf Extract can exert neuroprotective effects by stimulating TRPML1 and rescuing neuronal cells by boosting autophagy/mitophagy and upregulating a survival pathway^43^. A post-mortem study indicated that STN-DBS inhibited or reversed the reduction in mitochondrial volume and numbers caused by PD^18^. Similarly, in the present study, our results also indicated that STN-DBS could reduce mitochondrial injury. Our former study has revealed that STN-DBS can increase autophagy in SN^19^, nevertheless, some issues remain unknown, for instance, whether the mitophagy was influenced and through which pathway. PD patients display decreased activity of the complex I of the respiratory chain in the SN, along with mitochondrial damage and mitochondrial DNA deletions^14^. We showed STN stimulation reserved complex I activity in PD, which convinced us that the function of mitochondrion is preserved by this treatment. Mitophagy, the key process in keeping the cell healthy, promotes the turnover of mitochondria and prevents the accumulation of dysfunctional mitochondria which can lead to cellular degeneration. Therapies enhancing mitophagy and promoting the removal of damaged mitochondria are of interest for developing the disease management of PD. Small-molecule activators of PINK1 and parkin, or inhibitors of mTOR, USP30, and pSer65Ub phosphatases, are promising therapeutic targets for enhancing mitophagy in PD, and several of them have been found efficacious^44^. Particularly, mTOR-dependent mitophagy has been investigated widely. Similarly with other studies, we observed that MPTP injection could elevate the expression of mTOR^45^.

In some studies, both mTOR and p-mTOR were affected, however, in other studies, only p-mTOR was affected^46,47^. We found STN-DBS could suppress both mTOR and p-mTOR levels, suggesting that both the phosphorylation and expression of mTOR were affected, which resulted in the inhibition of the mTOR pathway. Meanwhile, an elevation of LC3 II with a decrease in p62 was observed in PD models treated with STN stimulation, combining the result of TEM and IF staining, it is believed that STN-DBS enhanced mitophagy in dopaminergic neurons via an mTOR-dependent manner.

Mitochondria are highly dynamic, undergoing fusion and fission ceaselessly, and damaged mitochondria are removed by mitophagy. Before fission, damaged DNA and proteins are segregated to one side of the mitochondrion, which is targeted for mitophagy, while the other daughter mitochondrion is now pristine^48^. The mitochondrial homeostasis, including fusion and fission cycles, is influenced in PD, and could be mediated by a set of energy-sensing factors (e.g., mTOR, AMPK, Sirtuins)^49^. In our study, we found a decrease in Drp1 and an increase in Opa1 were observed in PD models with stimulation of the STN, showing that mitochondrial fission and fusion were suppressed and elevated, respectively, compared to PD without stimulation. In other words, mitochondrial homeostasis was stabilized by STN-DBS.

Some studies have focused on the effect of genetic variability on PD patients with DBS. A claim that LRRK2 G2019S patients have greater improvement following surgery for STN-DBS than idiopathic patients was made in a comparative study^50^. Twelve months postoperatively, patients with parkin mutations had significantly lower levodopa equivalent daily dose than mutation-negative patients^51^, and patients with parkin mutations could be suitable for early surgery^52^. We found STN-DBS could increase the mitophagy in dopaminergic neurons in PD, which may illustrate why patients with mitophagy-related mutation showed positive outcomes to some degree.

Oxidative stress refers to “an imbalance between the generation of oxidants and their elimination systems, e.g., antioxidants in favor of oxidants, leading to disruption of redox signaling and control and/or molecular damage”^53^. Mitochondria are a major source of ROS as a byproduct of electron transport chain activity^49^. Impaired mitochondrial function results in a reduction in cellular energy and excessive ROS production in neurons, which in turn exacerbate mitochondrial damage^14^. Several markers of oxidative stress are down-regulated in the SN, CSF, and blood of PD patients, including oxidized DNA bases, coenzymes, and lipids, with a concomitant reduction of antioxidant molecules^54^. Overexpression of α-synuclein indicated that it interacts with several outer mitochondrial membrane components, including TOMM20 and VDAC, causing mitochondrial permeability transition pore opening, and oxidative stress^44,55,56^. In fact, dopaminergic neurons are extremely susceptible to oxidative stress, because numerous oxidants are produced when dopamine is released from synaptic vesicles into the synaptic cleft or the cytosol^14^. Antioxidants may act as scavengers of oxidants to maintain the biological redox steady states. Vitamin C and SOD, the major antioxidants, were significantly lower in patients with severe PD^57^. Mutation of the LRRK2, G2019S, could reduce mitophagy in PD, which can promote ROS-induced dopaminergic neuronal death, and the application of truncated LRRK2 reverses ROS accumulation and prevents neuronal injury^58^. We observed antioxidant enzymes, including SOD and GSH, were elevated by stimulation of STN, suggesting oxidative stress was relieved. However, when interrupted with an mTOR activator, this phenomenon disappeared, which demonstrates that the “antioxidant” role of STN-DBS relied on the mTOR signaling pathway.

Once permeabilization in the mitochondrial outer membrane is increased, several mitochondrial apoptogenic factors, including cytochrome c, second mitochondrial activator of caspases, and AIF, are released into the cytosol. In the cytosol, a multimeric structure composed of cytochrome c, apoptotic peptidase activating factor 1, and procaspase-9 is formed, which activates caspase-9, and then caspases-3, finally, this process results in apoptosis^14,54,59^. Researchers have tried to investigate novel therapies that could exert an antioxidative effect. For instance, Cu_x_O nanoparticle clusters functionally mimicked the activities of peroxidase, SOD, catalase, and glutathione peroxidase, which inhibits neurotoxicity and rescues memory loss in a PD model^15^. In the present study, the oxidative stress was suppressed by STN-DBS with elevated levels of antioxidant enzymes, and the mitochondrial apoptogenic factors, including AIF and cytochrome c, were released less from damaged mitochondria because mitochondrial function and structure were preserved, which resulted in less expression of cleaved-caspase 3 and 9. These findings convinced us that STN-DBS suppressed mitochondrial apoptogenic factors, which reduces cell apoptosis and exerts a neuroprotective effect. ROS bursts are also a pro-inflammatory stimulus via activation of nuclear factor κB^60^. Hou et al. found inhibition of NADPH oxidase via apocynin, alleviated impairments of learning and memory via the suppression of oxidative stress and neuroinflammation in PD^61^. Our former study showed STN-DBS could exert an anti-neuroinflammation effect via a nuclear factor κB dependent pathway^41^. It is hypothesized that the antioxidative effect of STN-DBS may reduce neuroinflammation.

In the present study, we only investigated the effect of a single stimulation frequency (130 Hz) on the motor performance and oxidative stress. Some researchers found a higher stimulation frequency (>130 Hz) may achieve the higher number of rotation in rat^62^. The stimulation frequency (130 Hz) in this study was selected based on the former studies^63–65^. And Isaac R. Cassare et al. found sporadic spike failure at high frequencies limits the efficacy of the informational lesion, yielding a parabolic profile with optimal effects at 130 Hz. However, we hope that the effect of the higher (>130 Hz) and lower (<100 Hz) stimulation frequency on the motor symptoms and oxidative stress will be investigated in the further study.

In conclusion, our study demonstrated that STN-DBS was able to increase mitophagy in dopaminergic neurons via an mTOR-dependent pathway, and oxidative stress was suppressed due to removal of damaged mitochondria. Consequently, apoptogenic factors released from mitochondria were reduced, which finally was attributed to the dopaminergic neuroprotection of STN-DBS in the SN of PD. These findings were confirmed in a variety of experiments in rodents, NHPs, and humans. Although an antioxidative effect was observed in PD patients with STN-DBS, a relationship between antioxidant levels and the therapeutic outcome was not obtained. Our study further contributes to understanding the mechanism of STN-DBS in controlling symptoms and inhibiting the disease progression of PD.

## Methods

### Animals, participants, and ethics

Adult (n = 379, 10-weeks old) male C57BL/6J mice and rhesus monkeys (*n* = 24, 6–9 years old) were provided by Beijing HFK Bioscience Co. Ltd. (Beijing, China) and the Laboratory Animal Center of the Military Medical Science Academy of China (Beijing, China), respectively. This study was approved by the Ethics Committee of Beijing Neurosurgical Institute (Process No. 202101017; 201704005) and was consistent with the National Institutes of Health Guide for the Care and Use of Laboratory Animals. To evaluate the motor impairment and dopaminergic neuron loss in a PD model and select the lead implantation time point, mice were assigned to control, PD-one (PD-1w), -two (PD-2w), -three (PD-3w), -four (PD-4w), -five (PD-5w), and -six (PD-6w) week groups. To investigate the effect of STN-DBS on mitophagy-mediated oxidative stress, mice were divided into control, PD, PD-sham-DBS, PD-DBS, PD+Rap, and PD-DBS + 3BDO groups. The monkeys were assigned to control, PD, PD-sham-DBS, and PD-DBS groups.

Eight patients (six females and two males; aged 65.9 ± 6.0 years old) with PD who were scheduled to undergo DBS surgery at Beijing Tiantan Hospital were recruited prospectively from 2019 to 2021, after approval by the institutional review board of Beijing Tiantan Hospital (KY 2018-008-01)^66^. All patients provided written informed consent.

### PD model establishment

The PD model was established using MPTP. For the mouse PD model, MPTP hydrochloride (25 mg/kg in saline, subcutaneous, Sigma–Aldrich, St. Louis, MO, USA) and probenecid (250 mg/kg in Tris-HCl buffer, Sigma–Aldrich) were administered over 4 weeks at 3.5-day intervals^67^. The animals in the control group received a normal saline injection of the same dosage. The mice in PD+Rap and PD-DBS + 3BDO were injected with rapamycin (7.5 mg/kg/day, 7 days, Selleck Chemicals, Houston, TX, USA) and 3BDO (80 mg/kg/day, 7 days, Selleck Chemicals), respectively, four weeks after the first MPTP injection. For the monkey PD model, all animals were given general anesthesia with intramuscular injection of Zoletil (5 mg/kg, Virbac, Alpes-Maritimes, France) and Dexdomitor (20 μg/kg, Zoetis, NJ, USA), before being fixed on the bed of a digital subtraction angiography (DSA) in a supine position. The left femoral artery was punctured using the Seldinger method. The left internal carotid artery was catheterized, and 20 ml saline containing MPTP (0.4 mg/kg) was pumped (with a constant speed) over a 20 min period^68,69^. The monkeys in the control received a normal saline injection of the same dosage.

### STN-DBS implantation

Four weeks after the first MPTP injection, mice in the PD-sham-DBS, PD-DBS, and PD-DBS + 3BDO groups were anesthetized (isoflurane, inhalation, RWD Life Science, Shenzhen, China) and prepared for the STN-DBS implantation. The concentric bipolar stimulation electrode was implanted in the left STN (AP = −2.06 mm, ML = +1.5 mm, DV = −4.5 mm) in these groups^70^. The electrodes of the PD-DBS and PD-DBS + 3BDO groups were connected to a stimulator (Master-8 Programmable Stimulator; AMPI, Jerusalem, Israel) that delivered an electrical pulse (frequency = 130 Hz, pulse width = 90 μs, intensity = 100 μA) for one week, but the electrodes in the PD-sham-DBS group were not connected to the stimulator. The other groups of animals did not receive the electrode implantation. After one week of stimulation, all mice were sacrificed. The SN (Only the ipsilateral SN; Due to technical difficulties, the SNpc and SNr regions were collected together.) of the mice was collected and stored at −80 °C and the rest of the mice was perfused with normal saline followed by 4% paraformaldehyde in 0.1 mol/L phosphate-buffered saline (PBS).

For NHP STN-DBS implantation, details were described in our previous study^24^. Briefly, the monkeys in the PD-sham-DBS and PD-DBS groups were anesthetized and underwent MRI (including 3D T1-, T2-weighted imaging, and magnetic resonance angiography) with a 3-Tesla MRI scanner (SIGNA; GE Healthcare, Waukesha, WI, USA). Six weeks after the first MPTP injection, the DBS leads (L301; Beijing PINS Medical Co. Ltd., Beijing, China) were used to target the left STN, according to individual MRI and the atlas of the rhesus monkey brain^71^. Electrode implantation was conducted by a neurosurgical robotic system, the accuracy of this new method was confirmed by our previous study^24,72^. An extension was tunneled subcutaneously from the neck to the abdomen where the implantable pulse generator (IPG, G102; Beijing PINS Medical Co. Ltd.) was located. The surgical complications and accuracy of lead placement were measured via postoperative CT. Another two weeks later, in the monkeys of the PD-DBS group, CT (fusion with MRI) was performed to select the optimal contact within the STN, and an electrical pulse (1.5 V, 90 μs and 130 Hz) was delivered through the selected contact (contact-, IPG + ). The monkey tissues (SN: SNpc + SNr) were collected two months after stimulation.

The patients with PD underwent bilateral STN-DBS through surgical procedures that were described in our previous studies^66,73^. Before surgery, the patients underwent a preoperative MRI and CT scan. Bilateral STN implantations were performed using a Leksell G frame system (Elekta Instrument AB, Stockholm, Sweden) under the guidance of preoperative images. Micro-electrode recordings as well as macro-stimulation were applied during surgery. The quadripolar DBS electrodes (Model L301; PINS Medical Co. Ltd., Beijing, China) were implanted and fixed and connected to the IPG. The patients were asked to come back to the hospital and received regular programming to achieve a satisfactory clinical outcome 4–5 weeks after surgery. The CSF of patients was collected preoperatively and 6 months after surgery and centrifuged at 4000 × g for 10 min at 4 °C.

### Behavior test

The rotarod test was used to evaluate motor deficits in the mice. Animals were pre-trained on an automated four-lane rotarod (Panlab, Harvard Apparatus, Barcelona, Spain) unit with a 3-cm diameter rod and an acceleration of 4 to 40 rpm over a period of 5 min, prior to MPTP injection. The length of time that each animal was able to stay on the rod was recorded as the latency to fall, which was automatically registered by a trip switch under the floor of the rotating drum. To evaluate the monkey motor impairment, the monkey hemiparkinsonism rating scale was used according to a previous study (Supplementary Table 1)^69^. Furthermore, contralateral rotation was measured following subcutaneous injection with APO (0.3 mg/kg) and recorded for 5 min, with a higher rotation number indicating a more severe motor impairment. The motor symptoms of PD patients were measured pre-operation and 6 months post-operation via the MDS-UPDRS III.

### Nissl staining

To evaluate the lead position in mice brains, frozen serial coronal sections (20 μm) of the brain containing the STN were cut and subjected to Nissl staining as previously described^8^. Briefly, slides containing the sections were sequentially immersed for 5 min in xylene followed by 100%, 95%, and 70% ethanol, then dipped in distilled water and stained with 0.5% cresyl violet solution for 15–30 min. After rinsing in water for 3–5 min and dehydrating in 70%, 95%, and 100% ethanol, the slides were placed in xylene for 10 min and the sections were covered with a coverslip.

### Western blot analysis

The left SN (SNpc + SNr) was washed with ice-cold PBS and lysed in radioimmunoprecipitation assay buffer composed of 50 mM Tris-HCl (pH 7.4), 150 mM sodium chloride, 1% Nonidet P-40, and 0.1% sodium dodecyl sulfate (SDS) containing phosphate and protease inhibitor cocktails. Homogenates were centrifuged at 12,000 × *g* for 20 min. The protein concentration in the supernatant was determined with a bicinchoninic acid protein assay kit (Pierce, Rockford, IL, USA) according to the manufacturer’s instructions. A total of 60 μg protein was resolved by SDS-polyacrylamide gel electrophoresis on an 8–12% polyacrylamide gel and transferred to a polyvinylidene difluoride membrane (Millipore, Billerica, MA, USA). After blocking with 10% milk for 1 h, the membrane was incubated with primary antibodies, including TH (T2928, Sigma–Aldrich, 1:2000), cleaved-caspase-3 (9661 s, Cell Signaling Technology, MA, USA, 1:1000), β-actin (A5060, Sigma–Aldrich, 1:5000), LC3 (L7543, Sigma–Aldrich, 1:1000), cleaved-caspase-9 (9507 s, Cell Signaling Technology, 1:1000), p62 (ab56416, Abcam, Cambridge, MA, USA 1:1000), Drp-1 (ab184247, Abcam, 1:1000), Opa-1 (ab157457, Abcam, 1:1000), cytochrome c (ab133504, Abcam, 1:5000), AIF (ab1998, Abcam, 1:1000), VDAC (4661 s, Cell Signaling Technology, 1:1000), mTOR (4517 s, Cell Signaling Technology, 1:1000), p-mTOR (5536 s, Cell Signaling Technology, 1:1000), following by incubated with secondary antibody, and protein bands were visualized by enhanced chemiluminescence; the signal intensity was quantified using ImageJ software. β-actin or VDAC was used as a control.

### Enzyme-linked immunosorbent assay (ELISA)

Levels of SOD (Cu/Zn cytosolic and Mn Mitochondrial SOD), GSH, and complex I activity in brain tissue and CSF were evaluated using ELISA according to the manufactory’s manual (SES134, CEA294Ge, Uscn Life Science Inc., Wuhan, China; ab136809, Abcam). Briefly, the tissue homogenates were centrifuged for 5 min at 10,000 × *g*, and supernatants were collected. Standard and diluted samples were added to separate wells in the reaction plates. After washing, a developing solution (provide by the ELISA kit) was added and the reaction was terminated with a stop solution. The optical density (OD) at 450 nm was measured with a microplate reader.

### IF staining and immunohistochemistry

For immunohistochemistry staining, the brain was removed and dehydrated in 20% and 30% sucrose solution, then cut into sections at a thickness of 40 μm, rinsed in PBS, and incubated in 3% H_2_O_2_ to quench endogenous peroxidase activity. After washing with PBS, sections were incubated in 10% goat serum followed by 0.3% TritonX-100 in PBS, then subsequently by overnight incubation at 4 °C with anti-TH antibody (T2928, Sigma–Aldrich, 1:2000) in a humidified chamber. Immunoreactivity was detected with a biotinylated secondary antibody (Zhongshan Golden Bridge Biotechnology Co., Beijing, China) and diaminobenzidine.

The number of TH^+^ neurons in each section was counted via unbiased stereology with Stereo Investigator software (MBF Biosciences, Williston, VT, USA). Consecutive sections from each brain (every sixth section, 40 μm) were selected throughout the entire rostrocaudal extent of the SNc for examination. Using the optical fractionator principle, the SN was outlined on each section at 5× magnification and TH^+^ neurons on the left sides were separately counted at 20× magnification under a brightfield microscope (Olympus, Tokyo, Japan). The weighted section thickness was used to correct for variations in tissue thickness at different sites.

The OD of TH^+^ neurites in the striatum (whole striatum) was determined using ImageJ software. The left striatum was selected as the measurement area. The OD of this area was measured and corrected by subtracting non-specific background signal. The average OD (OD/area) value was calculated.

For IF staining, the SN sections (20 μm) were rinsed in PBS and permeabilized with 0.3% Triton X-100 in PBS for 10 min at room temperature. After blocking with 10% normal goat serum for 1 h, sections were incubated overnight at 4 °C with primary antibodies, including p-mTOR (1:100, 5536 s, Cell Signaling Technology), TH (ab76442, Abcam, 1:1000; T2928, Sigma–Aldrich, 1:1000), TOMM20 (ab56783, Abcam, 1:1000), LC3 (L7543, Sigma–Aldrich, 1:100) followed by Alexa Fluor 647-, 594- or 488-conjugated secondary antibody (1:500; Life Technologies, Carlsbad, CA, USA) for 1 h at room temperature. Cell nuclei were visualized by counterstaining with 4′,6-diamidino-2-phenylindole (DAPI, Sigma–Aldrich). Sections were mounted on slides with 70% glycerol, covered with coverslips, and visualized using a confocal microscope (880, Zeiss, Oberkochen, Germany; TCS SP8; Leica, Solms, Germany). Fifteen cells were randomly selected from three matched sections (five cells in each section), and then the IF intensity was measured in each cell via ImageJ software. The average value was set as the value of the individual.

### TEM

TEM was performed according to a previous study^74^. The SN was washed in 0.1 M PBS and stored in 2.5% glutaraldehyde in 0.1 M PBS until processed. The slices were washed in 0.1 M PBS, post-fixed in 1% osmium tetroxide in 0.1 M PBS for 2 h, and washed again in 0.1 M PBS. Ultrathin sections were observed using an electron microscope (JEM2100; JEOL, Japan), and were observed in ten random cells and visual fields from each sample, scored according to the criteria, and recorded as described previously (Supplementary Table 2)^74^. When the condition was considered to fall between two scores, an increment of 0.5 points was added to the score. If more than one neuron or mitochondrion was observed in one field, the average grade was recorded.

### Statistical analysis

Data are expressed as the means ± standard deviations (SD). One-way ANOVA followed by the Tukey post-hoc correction was used to analyze the statistical significance of differences among multiple groups. Two-way ANOVA was used to analyze the rotarod test at different time points and groups, followed by a Tukey post-hoc correction for multiple comparisons. Two sample t test was used to measure the difference of Euclidean error. A paired-T test was used to evaluate the changes in PD patients over different time points. Pearson correlation test was used to investigate the correlation between changes in antioxidant enzymes and symtoms. Data were analyzed and plotted using GraphPad Prism version 9.5 software (GraphPad Software, La Jolla, CA, USA). A *P* < 0.05 was considered significant.

### Supplementary information

Supplementary

Related Manuscript File

## Data Availability

The data that support the findings of this study are available from the corresponding author upon request.
