# Supplementary material for: Subthalamic nucleus deep brain stimulation alleviates oxidative stress via mitophagy in Parkinson’s disease
Source: NPJ Parkinsons Dis. 2024 Mar 6;10:52. doi: 10.1038/s41531-024-00668-4 (PMC10917786; doi:10.1038/s41531-024-00668-4)
Supplement: Supplementary file 1 — Supplementary [file 41531_2024_668_MOESM1_ESM.pdf]

Supplementary Materials for

Subthalamic nucleus deep brain stimulation alleviates oxidative stress via mitophagy in Parkinson's disease

Yingchuan Chen[1,3] *et al.*

Corresponding author:

Jianguo Zhang[1,2,3]: Email: jg.zhang@ccmu.edu.cn

Address: Neurosurgery Department, Beijing Tiantan Hospital, South Four Ring West Road No. 119, B district, Fengtai Dist, Beijing, 100070, China.

Tingting Du[2,3]: Email: tingting.du@ccmu.edu.cn

Address: Department of Functional Neurosurgery, Beijing Neurosurgical Institute, South Four Ring West Road No. 119, B district, Fengtai Dist, Beijing, 100070, China.

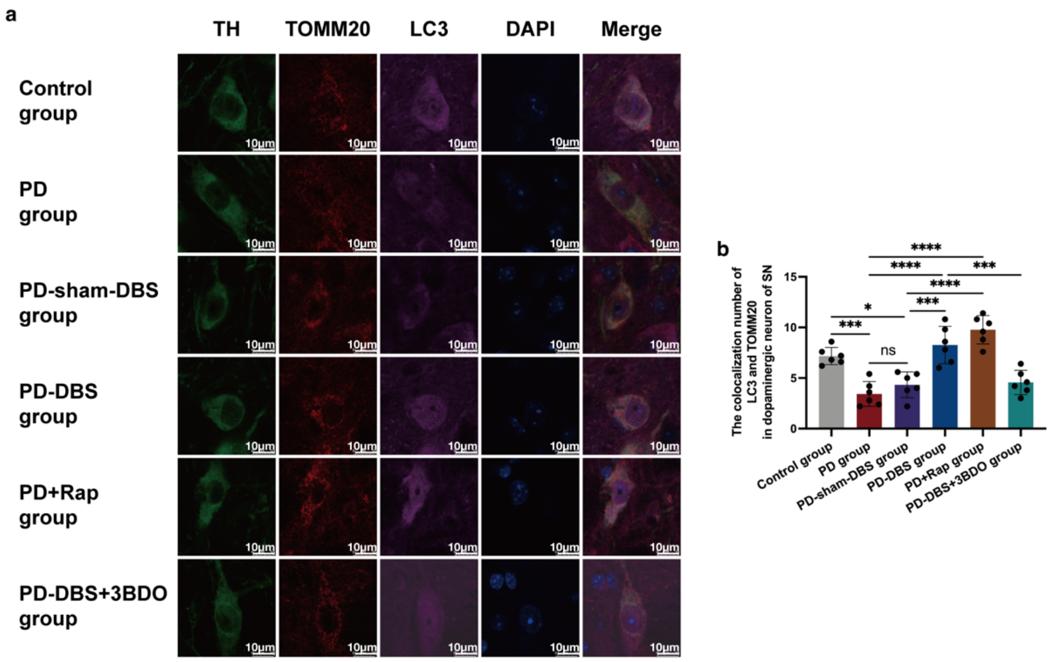

Supplementary Fig. 1. The mTOR-dependent mitophagy contributed to the neuroprotective effects of STN-DBS in PD mice. **(a).** Co-localization of TH, TOMM20, and LC3 in SN via IF staining (shared with the same mice in Fig. 3h). **(b).** The STN stimulation and rapamycin increased the co-expression of TOMM20 (mitochondria marker) and LC3 in the TH+ cell in the SN of the PD model, nevertheless, this could be interrupted by treatment with 3BDO (n = 6 per group; $F_{(5,30)} = 21.4$, $P < 0.0001$; one-way ANOVA followed by a Tukey post-hoc correction). $^{*}P < 0.05$; $^{**}P < 0.01$; $^{***}P < 0.001$; $^{****}P < 0.0001$. STN-DBS: subthalamic nuclei deep brain stimulation; PD: Parkinson's disease; SN: substantia nigra; IF: immunofluorescence; DAPI: 4′,6-diamidino-2-phenylindole; TH: tyrosine hydroxylase; ns: not significant.

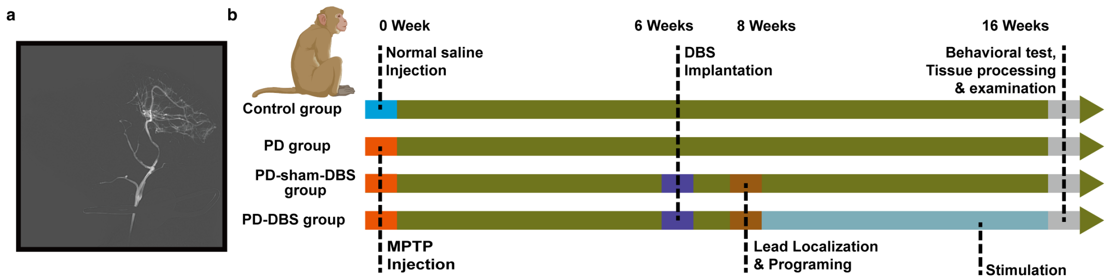

Supplementary Fig. 2. Chronic STN-DBS exerts an antioxidative role and stabilizes mitochondrial homeostasis in a PD monkey model. **(a).** MPTP internal carotid artery injection via DSA. **(b).** Experimental design of monkey STN-DBS. STN-DBS: subthalamic nuclei deep brain stimulation; PD: Parkinson's disease; MPTP: 1-methyl-4-phenyl-1,2,3,6-tetrahydropyridine; NS: normal saline; DSA: digital subtraction angiography.

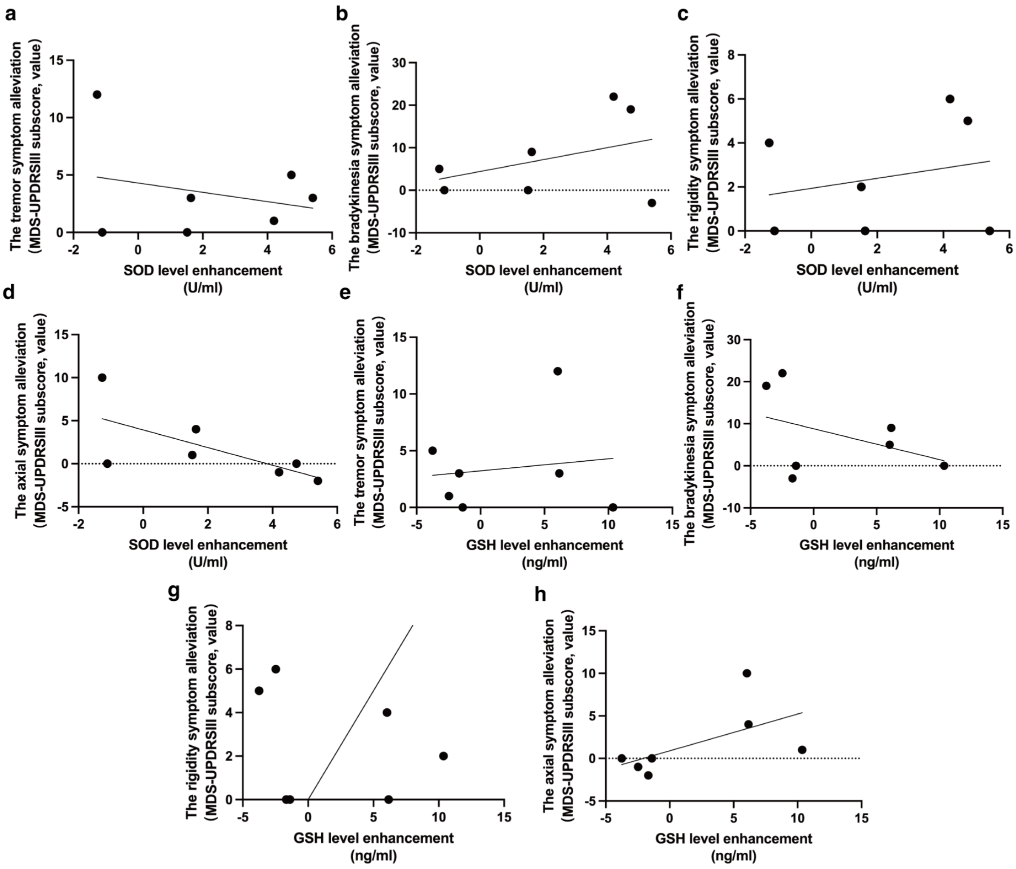

Supplementary Fig. 3. STN-DBS suppresses oxidative stress in PD patients. **(a–h).** no obvious correlation between changes in levels of SOD (tremor: *P*=0.5685; rigidity: *P*=0.6012; bradykinesia: *P*=0.3863; axial symptoms: *P*=0.0939) or GSH (tremor: *P*=0.7668; rigidity: *P*=0.5942; bradykinesia: *P*=0.3561; axial symptoms: *P*=0.1740), and alteration in tremor, rigidity, bradykinesia, and axial symptoms ($(Preoperative\ \mathrm{MDS-UPDRS})_{med-off} - (Postoperative\ MDS\ UPDRS)_{stm-on,med-off}$) (n = 7; Pearson correlation test). STN-DBS: subthalamic nuclei deep brain stimulation; PD: Parkinson's disease; MDS-UPDRS: Movement Disorder Society-Sponsored Revision of the Unified Parkinson's Disease Rating Scale; SOD: superoxide dismutase; GSH: glutathione.

Supplementary Table. 1. Monkey hemiparkinsonism rating scale.

**Tremor**	**Dyskinesia**
0=none	0=absent
1=some episodes	1=mild and intermittently present
2=frequent episodes	2=moderate and consistently present
3=persistent	3=severe and persistent, large amplitude
**Bradykinesia**	**Localization (if present)**
0=normal speed	0=contralateral
1=mild slowing of overall movements	1=ipsilateral
2=severe slowing of movements	2=both sides
3=akinesia (no movement)	
**Posture**	**Vomiting**
0=normal rising position	0=absent
1=flexed posture (0 ° - 45 °)	1=present
2=severely flexed posture (>90 °)	
3=dystonic posture	
**Rigidity***	**Overall level of activity**
0=none	+2=severe hyperkinesia; persistent excessive involuntary movement
1=mild	+1=moderate hyperkinesia; noticeable increase in involuntary movement
2=moderate	0=normal amount of movement
3=severe	-1=moderate hypokinesia; sparse movement
	-2=severe hypokinesia
**Arm movements (reaching for food outside of task) ***	
3=no movements	
2=sometimes	
1=frequent	
0=no differences with normal side	

*Only the contralateral side is quantified.

Supplementary Table. 2. Mitochondrial injury scale.

Score	Observation
0	Normal mitochondria (mitochondria appeared highly dense with well-organized cristae)
1	Early swelling as manifested by early clearing of matrix density and separation of cristae (large amorphous matrix density and linear density are present)
2	More marked swelling as manifested by further clearing of matrix density and separation of cristae
3	More extensive mitochondrial swelling with disruption of cristae
4	Severe mitochondrial swelling with disruption of cristae and rupture of inner and outer mitochondrial membranes

P values in "**STN-DBS relieved motor impairment and loss of dopaminergic neurons in the PD model**"

Rotarod test Adjusted P Value

Control group vs. PD group <0.0001

Control group vs. PD-sham-DBS group <0.0001

Control group vs. PD-DBS group 0.0246

Control group vs. PD+Rap group 0.0045

Control group vs. PD-DBS+3BDO group <0.0001

PD group vs. PD-sham-DBS group 0.9899

PD group vs. PD-DBS group 0.0038

PD group vs. PD+Rap group 0.021

PD group vs. PD-DBS+3BDO group 0.9695

PD-sham-DBS group vs. PD-DBS group 0.0204

PD-sham-DBS group vs. PD+Rap group 0.0918

PD-sham-DBS group vs. PD-DBS+3BDO group >0.9999

PD-DBS group vs. PD+Rap group 0.9891

PD-DBS group vs. PD-DBS+3BDO group 0.0318

PD+Rap group vs. PD-DBS+3BDO group 0.1328

TH-WB-SN Adjusted P Value

Control group vs. PD group <0.0001

Control group vs. PD-sham-DBS group <0.0001

Control group vs. PD-DBS group 0.0021

Control group vs. PD+Rap group 0.0421

Control group vs. PD-DBS+3BDO group <0.0001

PD group vs. PD-sham-DBS group 0.9994

PD group vs. PD-DBS group 0.0008

PD group vs. PD+Rap group <0.0001

PD group vs. PD-DBS+3BDO group 0.8639

PD-sham-DBS group vs. PD-DBS group 0.0003

PD-sham-DBS group vs. PD+Rap group <0.0001

PD-sham-DBS group vs. PD-DBS+3BDO group 0.6852

PD-DBS group vs. PD+Rap group 0.8385

PD-DBS group vs. PD-DBS+3BDO group 0.0159

PD+Rap group vs. PD-DBS+3BDO group 0.0007

TH-DAB-striatum Adjusted P Value

Control group vs. PD group <0.0001

Control group vs. PD-sham-DBS group <0.0001

Control group vs. PD-DBS group <0.0001

Control group vs. PD+Rap group <0.0001

Control group vs. PD-DBS+3BDO group <0.0001

PD group vs. PD-sham-DBS group >0.9999

PD group vs. PD-DBS group 0.143

PD group vs. PD+Rap group 0.0364

PD group vs. PD-DBS+3BDO group >0.9999

PD-sham-DBS group vs. PD-DBS group 0.1263

PD-sham-DBS group vs. PD+Rap group 0.0314

PD-sham-DBS group vs. PD-DBS+3BDO group >0.9999

PD-DBS group vs. PD+Rap group 0.9875

PD-DBS group vs. PD-DBS+3BDO group 0.0971

PD+Rap group vs. PD-DBS+3BDO group 0.023

TH-DAB-SN Adjusted P Value

Control group vs. PD group <0.0001

Control group vs. PD-sham-DBS group <0.0001

Control group vs. PD-DBS group <0.0001

Control group vs. PD+Rap group <0.0001

Control group vs. PD-DBS+3BDO group <0.0001

PD group vs. PD-sham-DBS group >0.9999

PD group vs. PD-DBS group 0.0074

PD group vs. PD+Rap group <0.0001

PD group vs. PD-DBS+3BDO group >0.9999

PD-sham-DBS group vs. PD-DBS group 0.0062

PD-sham-DBS group vs. PD+Rap group <0.0001

PD-sham-DBS group vs. PD-DBS+3BDO group 0.9998

PD-DBS group vs. PD+Rap group 0.5557

PD-DBS group vs. PD-DBS+3BDO group 0.0122

PD+Rap group vs. PD-DBS+3BDO group 0.0001

Original blot

Mice

Full unedited gel for Figure 1

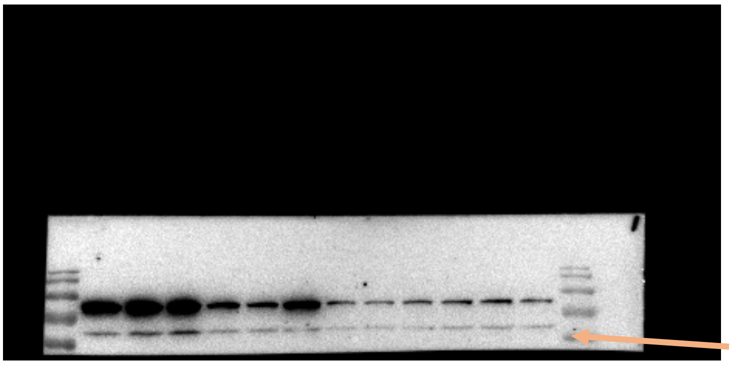

TH-str

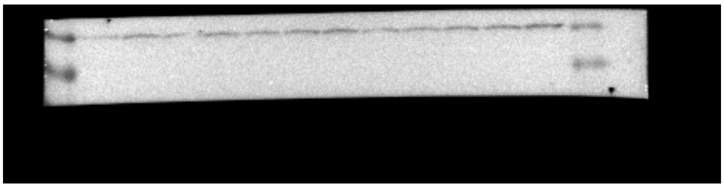

actin-str

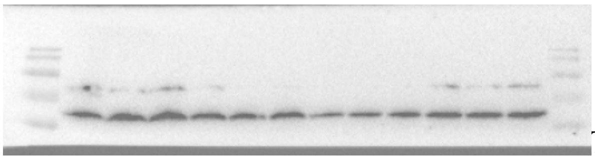

TH-sn

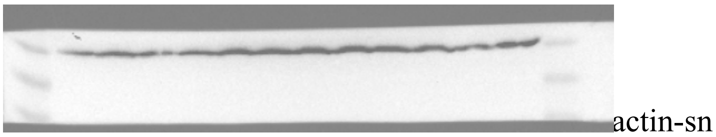

actin-sn

Full unedited gel for Figure 2

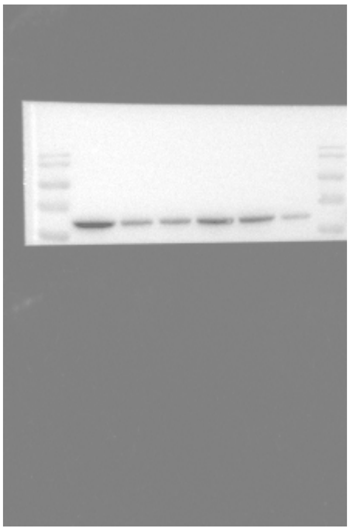

TH

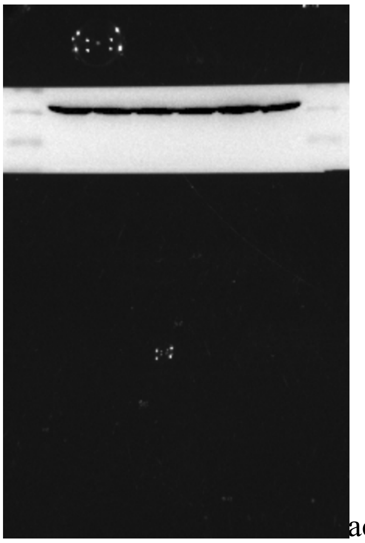
actin

Full unedited gel for Figure 3

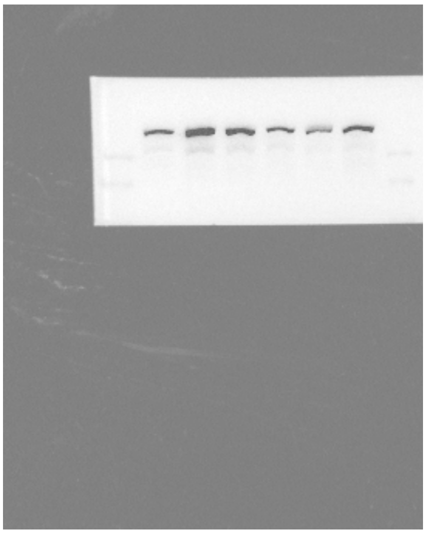
p-mTOR

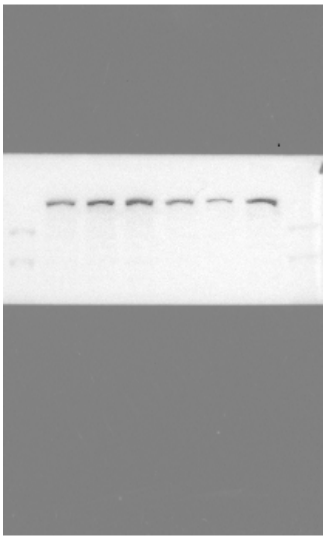

mTOR

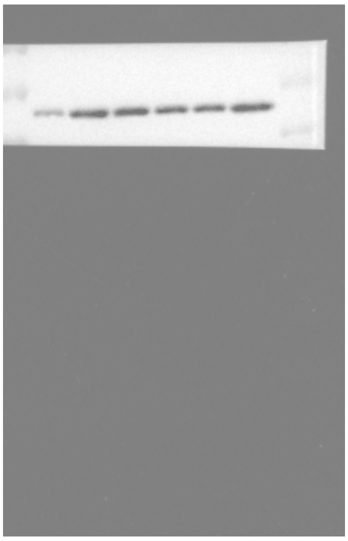

P62

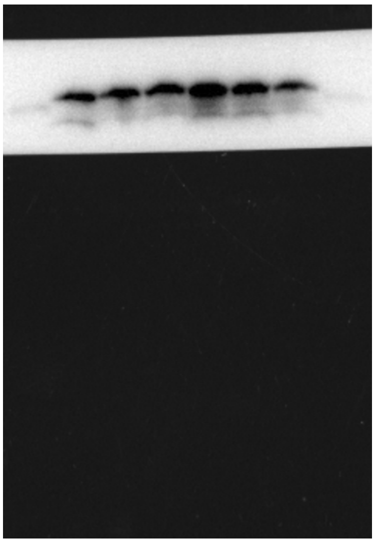

LC3

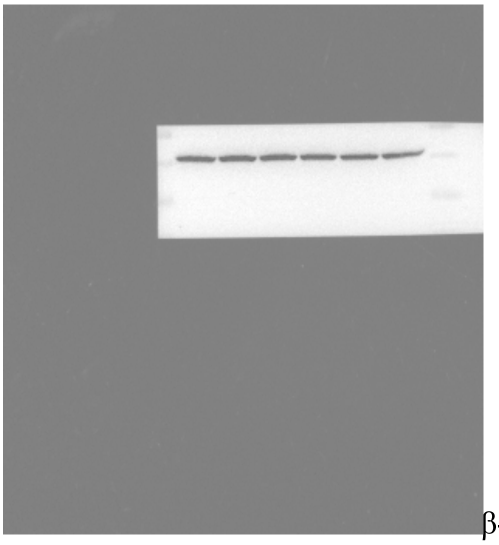

β-actin

Full unedited gel for Figure 4

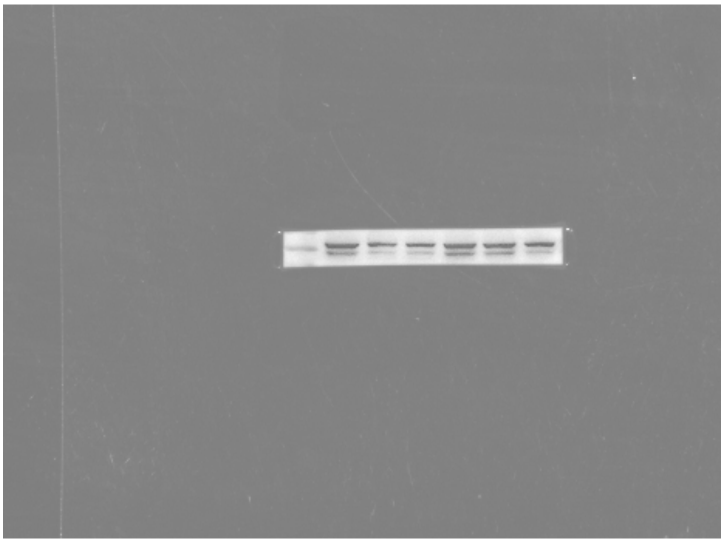

Opa1（mito）

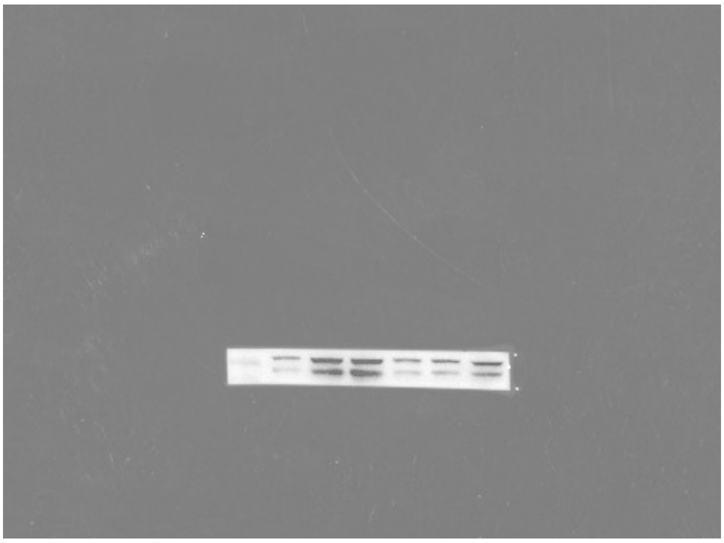

Drp1（mito）

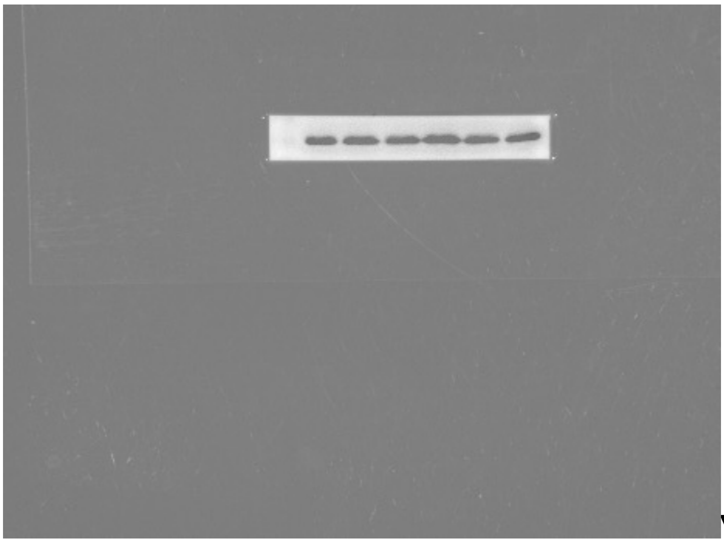

VDAC

Full unedited gel for Figure 5

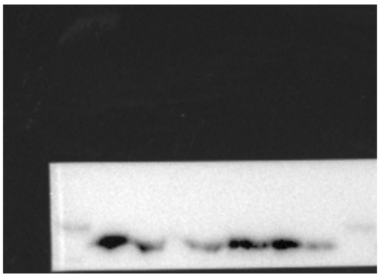

CytoC （mito）

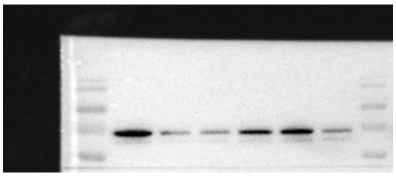

AIF（mito）

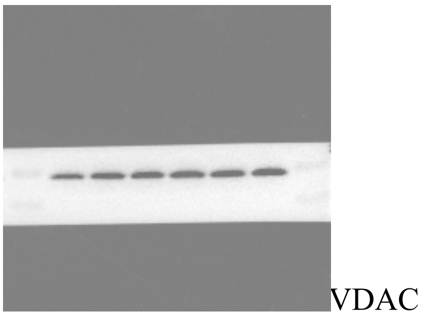

VDAC

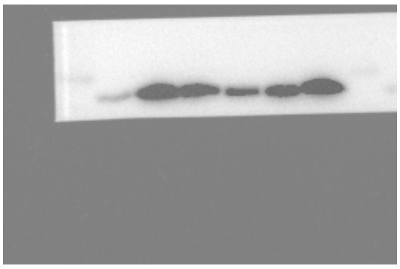

CytoC （cyto）

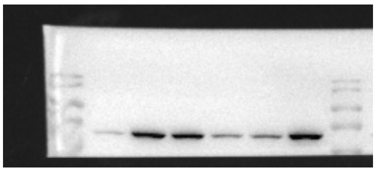

AIF （cyto）

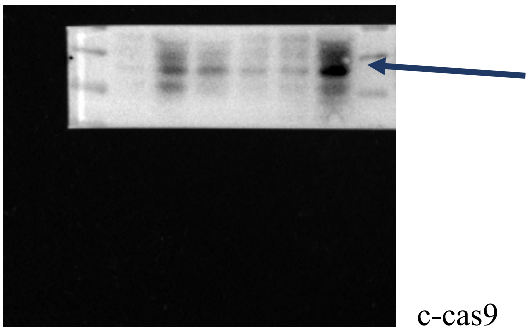

c-cas9

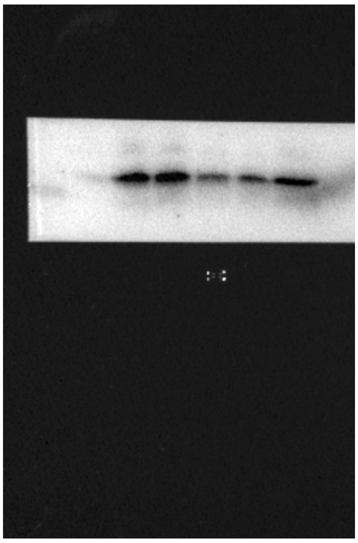

c-cas3

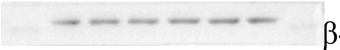

β-actin

Monkey western blot

Full unedited gel for Figure 6

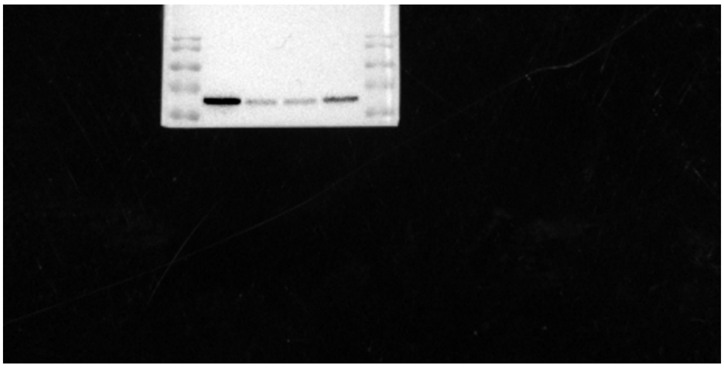

TH

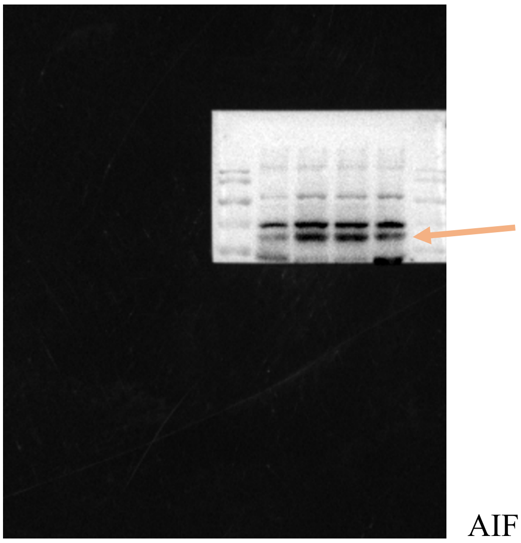

AIF（cyto）

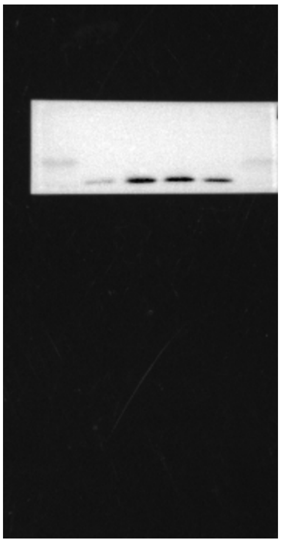

CytoC（cyto）

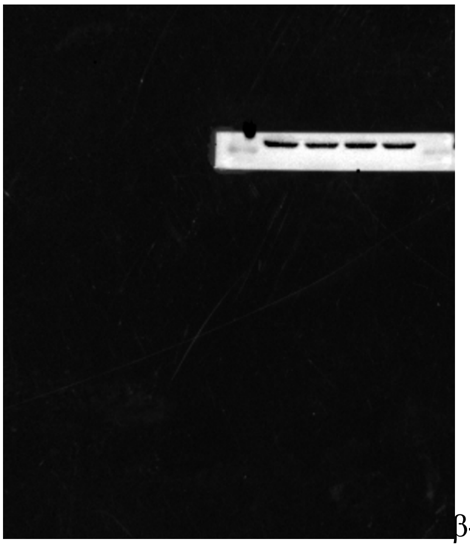

β-actin （cyto）

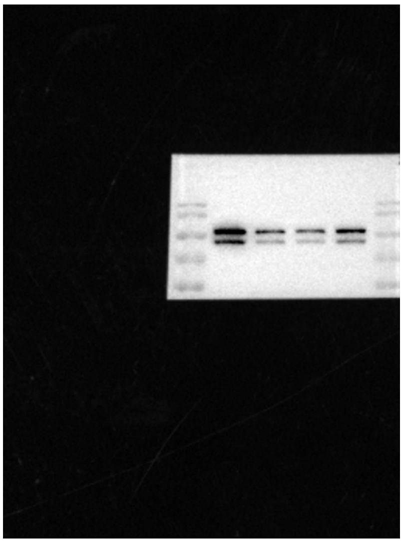

Opa1（mito）

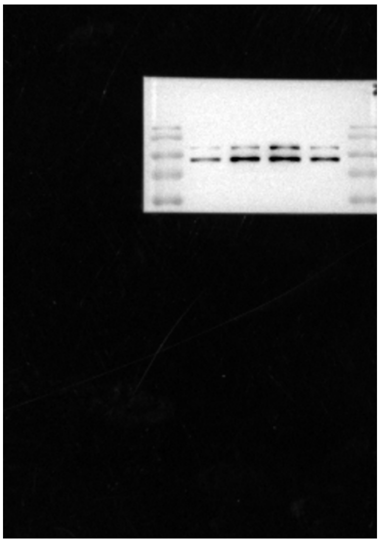

Drp1（mito）

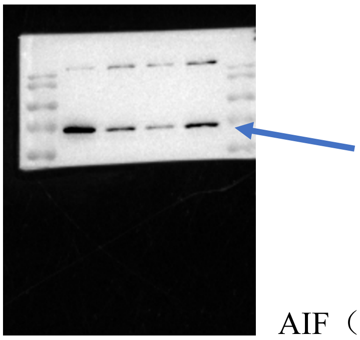

AIF（mito）

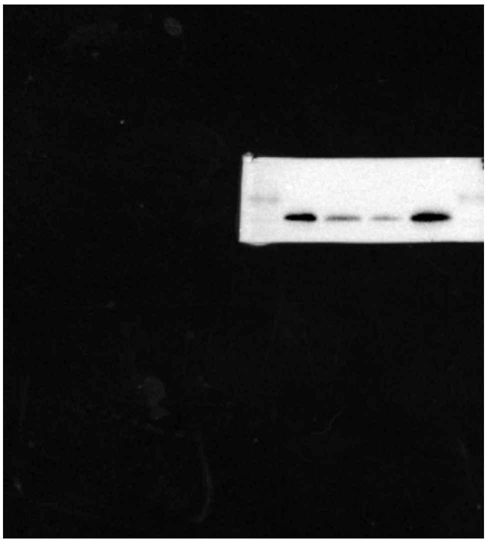

CytoC（mito）

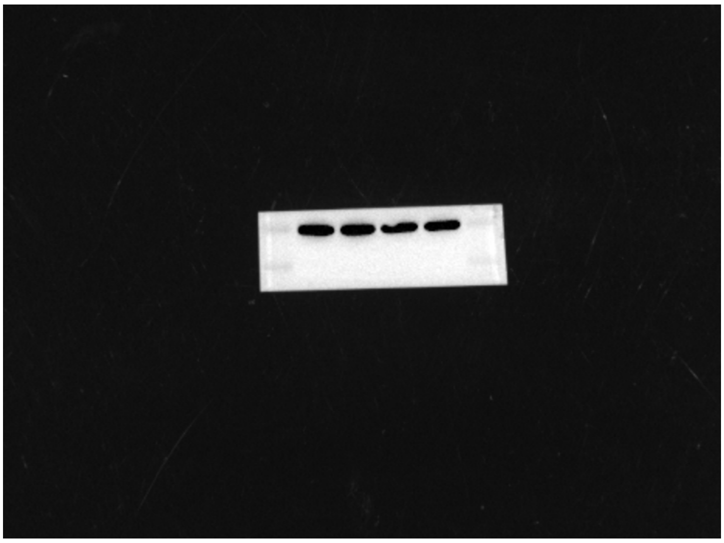

VDAC（mito）
