# Supplementary material for: Subthalamic nucleus deep brain stimulation alleviates oxidative stress via mitophagy in Parkinson’s disease
Source: NPJ Parkinsons Dis. 2024 Mar 6;10:52. doi: 10.1038/s41531-024-00668-4 (PMC10917786; doi:10.1038/s41531-024-00668-4)
Supplement: Supplementary file 2 — Related Manuscript File [file 41531_2024_668_MOESM2_ESM.pdf]

nature portfolio

Corresponding author(s): Jianguo Zhang

Last updated by author(s): Jan 31, 2024

Reporting Summary

Nature Portfolio wishes to improve the reproducibility of the work that we publish. This form provides structure for consistency and transparency in reporting. For further information on Nature Portfolio policies, see our Editorial Policies and the Editorial Policy Checklist.

Statistics

For all statistical analyses, confirm that the following items are present in the figure legend, table legend, main text, or Methods section.

n/a	Confirmed	
☐	☒	The exact sample size (*n*) for each experimental group/condition, given as a discrete number and unit of measurement
☐	☒	A statement on whether measurements were taken from distinct samples or whether the same sample was measured repeatedly
☐	☒	The statistical test(s) used AND whether they are one- or two-sided<br>*Only common tests should be described solely by name; describe more complex techniques in the Methods section.*
☐	☒	A description of all covariates tested
☐	☒	A description of any assumptions or corrections, such as tests of normality and adjustment for multiple comparisons
☐	☒	A full description of the statistical parameters including central tendency (e.g. means) or other basic estimates (e.g. regression coefficient) AND variation (e.g. standard deviation) or associated estimates of uncertainty (e.g. confidence intervals)
☐	☒	For null hypothesis testing, the test statistic (e.g. *F*, *t*, *r*) with confidence intervals, effect sizes, degrees of freedom and *P* value noted<br>*Give P values as exact values whenever suitable.*
☐	☒	For Bayesian analysis, information on the choice of priors and Markov chain Monte Carlo settings
☐	☒	For hierarchical and complex designs, identification of the appropriate level for tests and full reporting of outcomes
☐	☒	Estimates of effect sizes (e.g. Cohen's *d*, Pearson's *r*), indicating how they were calculated

Our web collection on statistics for biologists contains articles on many of the points above.

Software and code

Policy information about availability of computer code

Data collection	no computer code
Data analysis	no computer code

For manuscripts utilizing custom algorithms or software that are central to the research but not yet described in published literature, software must be made available to editors and reviewers. We strongly encourage code deposition in a community repository (e.g. GitHub). See the Nature Portfolio guidelines for submitting code & software for further information.

Data

Policy information about availability of data

All manuscripts must include a data availability statement. This statement should provide the following information, where applicable:

- Accession codes, unique identifiers, or web links for publicly available datasets
- A description of any restrictions on data availability
- For clinical datasets or third party data, please ensure that the statement adheres to our policy

Provide your data availability statement here.

Research involving human participants, their data, or biological material

Policy information about studies with human participants or human data. See also policy information about sex, gender (identity/presentation), and sexual orientation and race, ethnicity and racism.

Reporting on sex and gender	Yes
Reporting on race, ethnicity, or other socially relevant groupings	Yes
Population characteristics	Yes
Recruitment	Yes
Ethics oversight	Yes

Note that full information on the approval of the study protocol must also be provided in the manuscript.

Field-specific reporting

Please select the one below that is the best fit for your research. If you are not sure, read the appropriate sections before making your selection.

☒ Life sciences ☐ Behavioural & social sciences ☐ Ecological, evolutionary & environmental sciences

For a reference copy of the document with all sections, see nature.com/documents/nr-reporting-summary-flat.pdf

Life sciences study design

All studies must disclose on these points even when the disclosure is negative.

Sample size	8
Data exclusions	no
Replication	Yes
Randomization	No randomization
Blinding	No

Reporting for specific materials, systems and methods

We require information from authors about some types of materials, experimental systems and methods used in many studies. Here, indicate whether each material, system or method listed is relevant to your study. If you are not sure if a list item applies to your research, read the appropriate section before selecting a response.

Materials & experimental systems

n/a	Involved in the study	
☐	☒	Antibodies
☒	☐	Eukaryotic cell lines
☒	☐	Palaeontology and archaeology
☐	☒	Animals and other organisms
☐	☒	Clinical data
☒	☐	Dual use research of concern
☒	☐	Plants

Methods

n/a	Involved in the study	
☒	☐	ChIP-seq
☒	☐	Flow cytometry
☐	☒	MRI-based neuroimaging

Antibodies

Antibodies used	TH (T2928, Sigma–Aldrich, 1:2000), cleaved-caspase-3 (9661s, Cell Signaling Technology, MA, USA, 1:1000), β-actin (A5060, Sigma–Aldrich, 1:5000), LC3 (L7543, Sigma–Aldrich, 1:1000), cleaved-caspase-9 (9507s, Cell Signaling Technology, 1:1000), p62 (ab56416, Abcam, Cambridge, MA, USA 1:1000), Drp-1 (ab184247, Abcam, 1:1000), Opa-1 (ab157457, Abcam, 1:1000), cytochrome c (ab133504, Abcam, 1:5000), AIF (ab1998, Abcam, 1:1000), VDAC (4661s, Cell Signaling Technology, 1:1000), mTOR (4517s, Cell

	Signaling Technology, 1:1000), p-mTOR (5536s, Cell Signaling Technology, 1:1000),p-mTOR (1:100, 5536s, Cell Signaling Technology), TH (ab76442, Abcam, 1:1000; T2928, Sigma–Aldrich, 1:1000), TOMM20 (ab56783, Abcam, 1:1000), LC3 (L7543, Sigma–Aldrich, 1:100)
Validation	These antibodies have been validated by the manufactories

Animals and other research organisms

Policy information about studies involving animals; ARRIVE guidelines recommended for reporting animal research, and Sex and Gender in Research

Laboratory animals	mice and monkey
Wild animals	No
Reporting on sex	male
Field-collected samples	a standard enviorment
Ethics oversight	approved by the Ethics Committee

Note that full information on the approval of the study protocol must also be provided in the manuscript.

Clinical data

Policy information about clinical studies

All manuscripts should comply with the ICMJE guidelines for publication of clinical research and a completed CONSORT checklist must be included with all submissions.

Clinical trial registration	Yes
Study protocol	ChiCTR1900026601
Data collection	Data collection by researchers
Outcomes	Scale scoring

Plants

Seed stocks	No involved
Novel plant genotypes	No involved
Authentication	No involved

Magnetic resonance imaging

Experimental design

Design type	Structural MRI
Design specifications	No special design
Behavioral performance measures	Scale scoring

Acquisition

Imaging type(s)	T2 3DT1
Field strength	3.0
Sequence & imaging parameters	three-dimensional sagittal T1-weighted-3D magnetization-prepared rapid acquisition gradient echo (MPRAGE)

Sequence & imaging parameters	sequence (repetition time, 6.6 ms; echo time, 3.1 ms; flip angle, 8°; matrix size, 240 × 240; isotropic voxel, 1 × 1 × 1 mm3; number of slices, 196)
Area of acquisition	whole brain

Diffusion MRI ☐ Used ☒ Not used

Preprocessing

Preprocessing software	LeadDBS
Normalization	ANTs
Normalization template	MNI
Noise and artifact removal	no involved
Volume censoring	no involved

Statistical modeling & inference

Model type and settings	no involved
Effect(s) tested	no involved

Specify type of analysis: ☒ Whole brain ☐ ROI-based ☐ Both

Statistic type for inference (See Eklund et al. 2016)	no involved
Correction	no involved

Models & analysis

n/a	Involved in the study
☒	☐ Functional and/or effective connectivity
☒	☐ Graph analysis
☒	☐ Multivariate modeling or predictive analysis
